# Bypassing the Pentose Phosphate Pathway: Towards Modular Utilization of Xylose

**DOI:** 10.1371/journal.pone.0158111

**Published:** 2016-06-23

**Authors:** Kulika Chomvong, Stefan Bauer, Daniel I. Benjamin, Xin Li, Daniel K. Nomura, Jamie H. D. Cate

**Affiliations:** 1 Department of Plant and Microbial Biology, University of California, Berkeley, CA, United States of America; 2 Energy Biosciences Institute, Berkeley, CA, United States of America; 3 Program in Metabolic Biology, Department of Nutritional Sciences and Toxicology, University of California, Berkeley, CA, United States of America; 4 Department of Molecular and Cell Biology, University of California, Berkeley, CA, United States of America; 5 Department of Chemistry, University of California, Berkeley, CA, United States of America; 6 Physical Biosciences Division, Lawrence Berkeley National Laboratory, Berkeley, CA, United States of America; Korea University, REPUBLIC OF KOREA

## Abstract

The efficient use of hemicellulose in the plant cell wall is critical for the economic conversion of plant biomass to renewable fuels and chemicals. Previously, the yeast *Saccharomyces cerevisiae* has been engineered to convert the hemicellulose-derived pentose sugars xylose and arabinose to d-xylulose-5-phosphate for conversion via the pentose phosphate pathway (PPP). However, efficient pentose utilization requires PPP optimization and may interfere with its roles in NADPH and pentose production. Here, we developed an alternative xylose utilization pathway that largely bypasses the PPP. In the new pathway, d-xylulose is converted to d-xylulose-1-phosphate, a novel metabolite to *S*. *cerevisiae*, which is then cleaved to glycolaldehyde and dihydroxyacetone phosphate. This synthetic pathway served as a platform for the biosynthesis of ethanol and ethylene glycol. The use of d-xylulose-1-phosphate as an entry point for xylose metabolism opens the way for optimizing chemical conversion of pentose sugars in *S*. *cerevisiae* in a modular fashion.

## Introduction

Utilization of pentose sugars in hemicellulose is essential for economical biofuel and renewable chemical production from plant cell wall derived biomass [1,2]. Although highly efficient at fermenting glucose, most *Saccharomyces cerevisiae* strains cannot utilize xylose and arabinose [3]. To broaden its substrate spectrum, heterologous enzymes from bacteria and fungi have been successfully engineered into *S*. *cerevisiae* to enable xylose and arabinose consumption [3–5]. These pathways deliver xylose and arabinose to the endogenous pentose phosphate pathway (PPP) via d-xylulose-5-phosphate (X5P). It has been found that to improve pentose utilization efficiency, expression of the endogenous PPP enzymes must be manipulated [6,7]. This may be because the PPP in *S*. *cerevisiae* is primarily dedicated to NADPH regeneration and ribose 5-phosphate synthesis [8,9], not for xylose and arabinose utilization. A systematic approach to identify the limiting steps for pentose utilization via the PPP requires careful investigation of the regulation of many enzymes and metabolites in the PPP and in glycolysis. One proposed alternative involves the addition of a heterologous phosphoketolase pathway, producing ethanol via conversion of X5P to glyceraldehyde-3-phosphate and acetyl-phosphate [10]. However, the proposed system still relies on and produces X5P, a key intermediate metabolite in the PPP [10]. Here, we explored an alternative 3-step pentose utilization pathway, designed to bypass the endogenous PPP.

**Fig 1:**
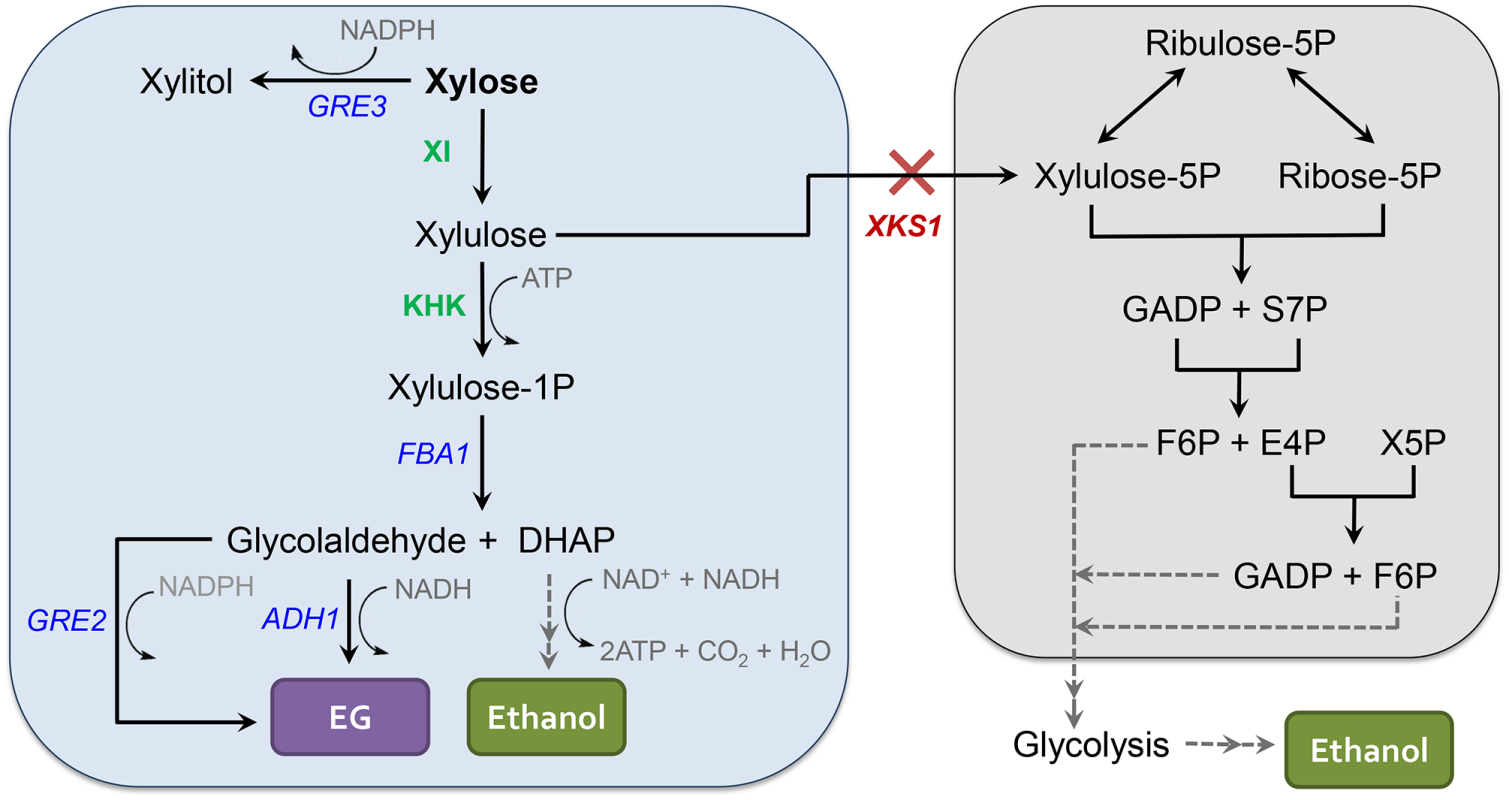
An alternative pentose sugar utilization in *S*. *cerevisiae*. The system consists of two exogenous components, XI and KHK, which convert d-xylose to d-xylulose and X1P, respectively. X1P is then metabolized by *S*. *cerevisiae* endogenous enzymes—*FBA1*, *GRE2* and/or *ADH1*—producing glycolaldehyde and DHAP as intermediates and EG and ethanol as final products. *XKS1* deletion allows the metabolic flux to be directed to the synthetic pathway via X1P by eliminating a possible route of xylulose utilization via X5P through the PPP. Abbreviations in the figure are: PPP, pentose phosphate pathway; XI, xylose isomerase; KHK, ketohexokinase; *FBA1*, fructose 1,6-bisphosphate aldolase; *GRE2*, 3-methylbutanal reductase; *ADH1*, alcohol dehydrogenase; *XKS1*, xylulokinase; *GRE3*, aldose reductase; EG, ethylene glycol; Xylulose-1P, xylulose 1-phosphate; Xylulose-5P/X5P, xylulose 5-phosphate; DHAP, dihydroxyacetone phosphate; GADP, glyceraldehyde 3-phosphate; S7P, sedoheptulose 7-phosphate; F6P, fructose 6-phosphate; E4P, erythrose 4-phosphate.

The alternative xylose utilization pathway in *S*. *cerevisiae* is comprised of 3 main steps (Fig 1). First, d-xylose is converted to d-xylulose by xylose isomerase (XI) or the two enzymes xylose reductase (XR) and xylitol dehydrogenase (XDH) [11–13]. d-xylulose is then phosphorylated to d-xylulose-1-phosphate (X1P) by an ATP-dependent ketohexokinase (KHK) [14,15]. The third step is catalyzed by endogenous fructose-1,6-bisphosphate aldolase (*FBA1*), which can cleave X1P to glycolaldehyde and dihydroxyacetone phosphate (DHAP) [14]. These three steps would deliver three carbons from xylose to glycolysis using DHAP as an intermediate. The second product from the third step, glycolaldehyde, is normally toxic to yeast [16], but could be converted to ethylene glycol (EG) by endogenous NADH-dependent alcohol dehydrogenase (*ADH1*) and/or NADPH-dependent 3-methylbutanl/methylglyoxal reductase (*GRE2*). The *in vivo* activities of these enzymes on glycolaldehyde in *S*. *cerevisiae* have been demonstrated [17,18]. The alternative pathway thus should enable xylose utilization in *S*. *cerevisiae*. A synthetic utilization of xylose via X1P has been demonstrated in *Escherichia coli* [19,20].

We reasoned that the proposed three-step xylose utilization pathway would likely require further engineering to alleviate potential inefficiencies. Endogenous xylulokinase (*XKS1*), which is responsible for phosphorylating d-xylulose to X5P, might direct most of the d-xylulose flux from xylose to the PPP. *XKS1* might therefore need to be disrupted so that the flux of d-xylulose is directed to the new synthetic pathway. In addition, the pathway as envisioned generates 1 net ATP and an excess of NAD^+^ and/or NADP^+^. Thus, the pathway may need to be supplied with additional ATP and reducing power to produce a functional and balanced system.

Here, we tested whether the new pathway functions in *S*. *cerevisiae*, allowing xylose utilization via an intermediate metabolite, X1P, typically not present in traditional pentose utilization.

## Results

### Construction of the synthetic xylose utilization pathway in *S*. *cerevisiae*

As a first test of the synthetic xylose utilization pathway (Fig 1), we used the XI from *Bacteroides stercoris* HJ-15 for the conversion of d-xylose to d-xylulose. We hypothesized that rat liver KHK might be used to catalyze the second step of the pathway–the conversion of d-xylulose to d-xylulose-1-phosphate (X1P)–due to its similarity to ketohexokinase from human liver (80% amino acid identity), which catalyzes this reaction [14], and the fact that rat liver KHK can phosphorylate fructose in *S*. *cerevisiae* cell lysates [15]. Finally, we relied on the endogenous fructose 1,6-bisphosphate aldolase (*FBA1*) to cleave X1P to glycolaldehyde and dihydroxyacetone phosphate (DHAP), as a partially purified aldolase from human liver could cleave X1P *in vitro* [14].

**Fig 2:**
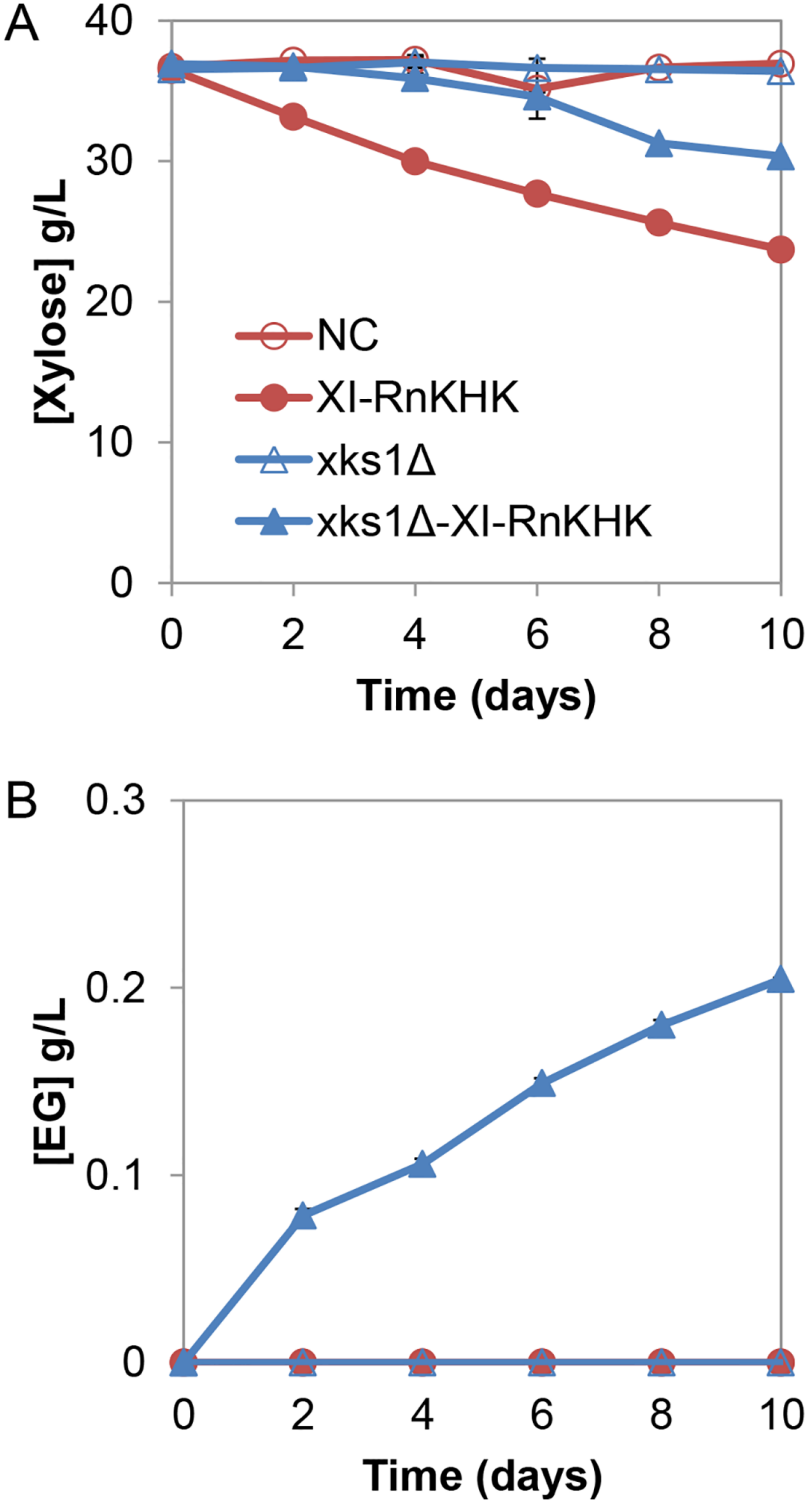
Comparisons of fermentation profiles using strain with and without *xks1Δ*, in the presence and absence of exogenous xylose isomerase (XI) and ketohexokinase (RnKHK) expression. Concentrations of **(A)** xylose and **(B)** ethylene glycol (EG) are shown. NC, negative control. Error bars indicated standard errors, N = 2.

Exogenous components of the synthetic pathway (Fig 1)–xylose isomerase (XI) from *B*. *stercoris* HJ-15 and ketohexokinase (RnKHK) from rat liver–were introduced into *S*. *cerevisiae* strain D452-2 and a D452-2 strain with xylulokinase deleted (D452-2 *xks1*Δ::KanMX) to eliminate xylulose conversion to X5P. To test xylose consumption by these strains, fermentation experiments with 40 g/L of xylose were carried out under strict anaerobic conditions. Xylose was consumed when XI and RnKHK were expressed in the backgrounds with and without *xks1*Δ (Fig 2A). However, xylose consumption in the *xks1*Δ background did not start until after approximately 6 days while that in the strain with functional *XKS1* started immediately (Fig 2A). In total, the amount of xylose consumed after 10 days in the *xks1*Δ background was approximately 2-fold lower than that with functional *XKS1* (Fig 2A). Notably, EG production was detected only when XI and RnKHK were expressed in the *xks1*Δ background (Fig 2B). These results suggest that *XKS1* deletion was necessary for driving metabolic flux through d-xylulose-1-phosphate to produce ethylene glycol. Nevertheless, the decrease in xylose consumption in the *xks1*Δ background implied that components downstream of the xylose isomerase step were potential bottlenecks of the pathway. Concentrations of xylitol and glycerol, which are byproducts of xylose metabolism, were lower in the *xks1Δ* background, consistent with a lower carbon flux passing through the pathway (S1 Fig). We also attempted to substitute XR and XDH for XI to convert xylose to xylulose. However, the resulting strain used xylose poorly and did not produce EG (S1 Fig) and thus was not pursued further.

### Overexpression of the endogenous components in the synthetic pathway

**Fig 3:**
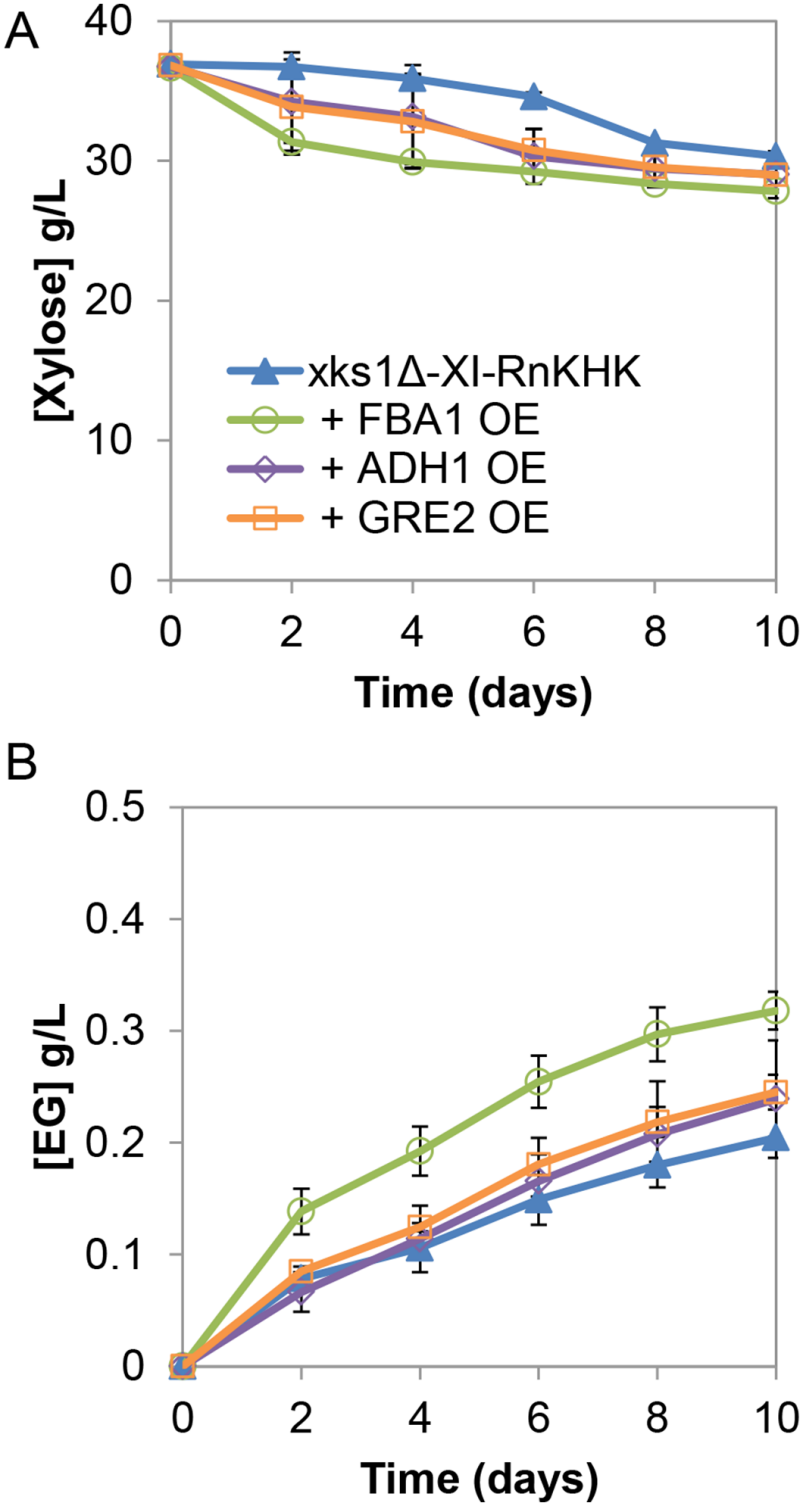
Effects of overexpression of endogenous enzymes in the alternative xylose utilization pathway. Strains overexpressing the three endogenous components–*FBA1*, *ADH1*, and *GRE2*—were compared. *xks1Δ* XI-RnKHK were used as the background strain for all the overexpression comparisons. Concentrations of **(A)** xylose and **(B)** ethylene glycol (EG) were shown. OE, overexpression. Error bars indicated standard errors, N = 2.

In addition to the two exogenous components shown to be necessary for EG biosynthesis, three endogenous enzymes involved in the synthetic pathway—*FBA1*, *GRE2* and *ADH1* (Fig 1)—were individually overexpressed in the *xks1*Δ XI-RnKHK strains. *FBA1* overexpression (OE) showed 33% and 56% increases in total amount of xylose consumed and EG titer, respectively (Fig 3). *FBA1* overexpression resulted in immediate xylose consumption in contrast to the approximately 6-day lag phase observed in the starting strain (Fig 3A). The positive impacts of *GRE2* and *ADH1* overexpression, while observable, were not as prominent as that of *FBA1* overexpression (Fig 3). No further improvements were observed when *GRE2* and/or *ADH1* were overexpressed along with *FBA1* (S2 Fig). Production of xylitol, glycerol and ethanol positively correlated with the amount of xylose consumed (S2 Fig). These results suggested that the pathway was primarily limited by the aldolase activity of Fba1, responsible for converting d-xylulose-1-phosphate to glycolaldehyde and DHAP, and not the final reduction of glycolaldehyde to ethylene glycol by Gre2p and/or Adh1.

### Cellobiose co-utilization enhanced the synthetic xylose consumption pathway

**Fig 4:**
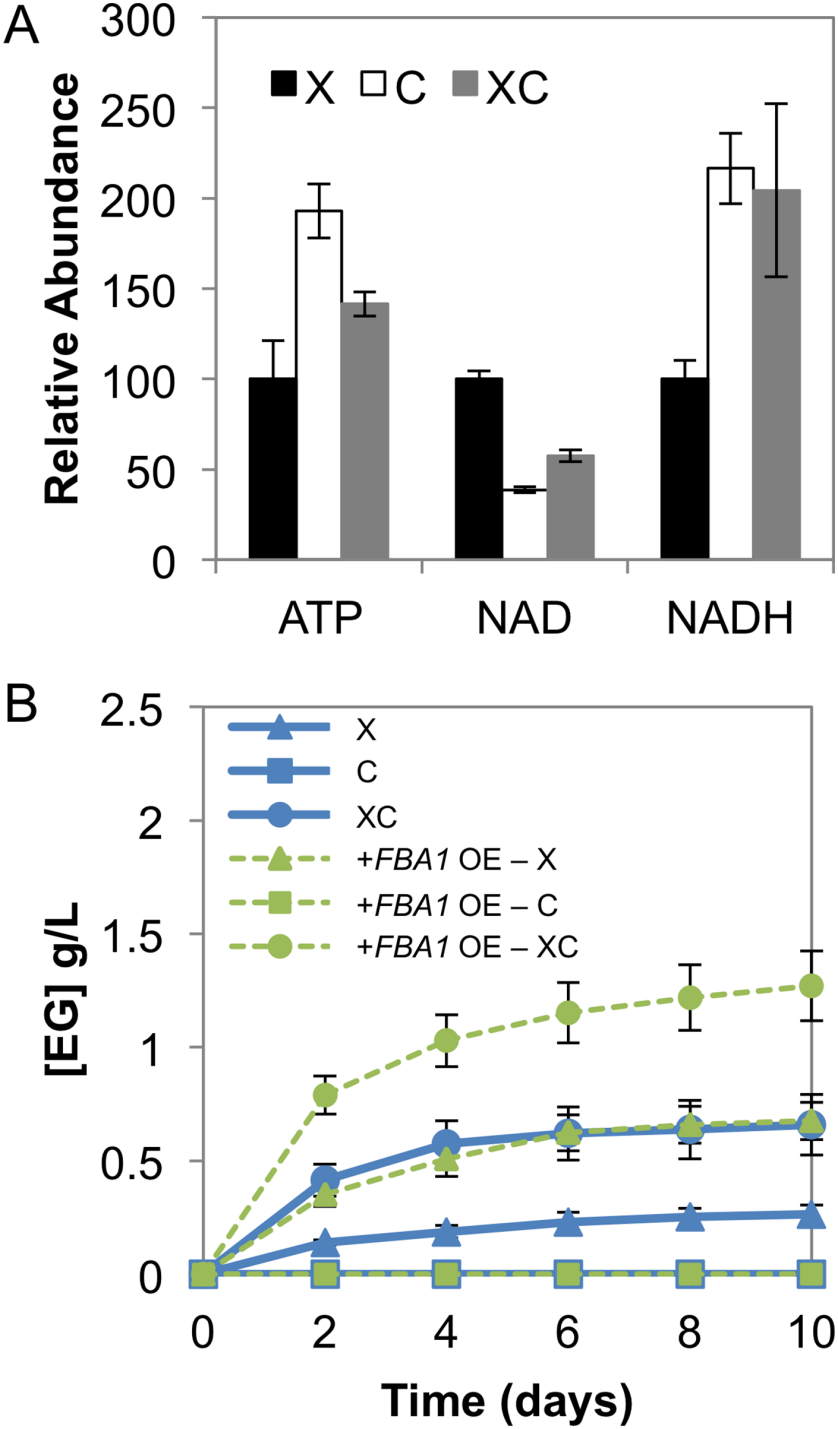
Synergistic effects of cellobiose and xylose co-utilization. **(A)** Intracellular concentrations of ATP, NAD^+^ and NADH are shown for fermentations with xylose, cellobiose and its mixture (denoted, X, C and XC respectively) provided to the *xks1Δ* XI-RnKHK-*FBA1*-CD strain. **(B)** *xks1Δ* XI-RnKHK-CD (denoted in blue) and *xks1Δ* XI-RnKHK-*FBA1*-CD (denoted in green) strains were provided with xylose in the presence and absence of cellobiose. Concentrations of ethylene glycol (EG) are shown. X, xylose; C, cellobiose; XC, mixture of xylose and cellobiose; OE, overexpression. Error bars indicated standard errors, N = 5.

Previous experiments demonstrated that a cellodextrin consumption pathway (CD), comprised of a cellodextrin transporter and intracellular β-glucosidase from *Neurospora crassa* [21], could be used for the co-consumption of cellobiose and xylose [22]. We reasoned that co-consumption of cellobiose might increase the ATP levels and NAD(P)H reducing equivalents available for the alternative xylose consumption pathway. In the co-utilization system, intracellular ATP and NADH were 42% and 104% higher than that provided with xylose as the sole carbon source (Fig 4A), as predicted. When cellobiose and xylose were co-utilized, ethylene glycol titers increased by 30% and 160% in strains expressing endogenous levels of *FBA1* or overexpressing *FBA1* (strains *xks1*Δ XI-RnKHK-CD and *xks1*Δ XI-RnKHK-*FBA1*-CD), respectively (Fig 4B). Co-utilization of cellobiose resulted in improved xylose consumption, increased xylitol production, increased glycerol production and improved cell viability (OD600) in comparison to when xylose was provided as a sole carbon source (S3 Fig). Compared to cellobiose consumption as the sole carbon source, a 15% decrease in glycerol titer was observed (S3 Fig). These results show that cellobiose co-utilization enhanced xylose consumption via the synthetic pathway, by supplying the system with excess ATP and NADH. Furthermore, they revealed that cellobiose co-utilization and *FBA1* overexpression had a synergistic effect on the efficiency of the xylose consumption pathway.

We further investigated whether supplying additional NADH in the absence of cellobiose could increase ethylene glycol production. The engineered yeast (*xks1*Δ XI-RnKHK-*FBA1*) were provided with xylose in anaerobic fermentations in the presence of formate, which is the substrate for endogenous NADH-generating formate dehydrogenase (Fdh1) similar to NADH-generating butanediol dehydrogenase (Bdh1) [23]. The activities of Fdh1 were verified in crude cell lysates, which were incubated with NAD^+^ and formate (S4A Fig). When the strains were provided with 25 mM formate, a decrease in formate concentration was detected. In the presence of formate, improved xylose consumption, decreased EG, xylitol and glycerol productions were detected while ethanol production remained unchanged (S4 Fig). These results imply that an attempt to provide excess NADH alone may help improve xylose consumption but is not sufficient to improve carbon conversion by the synthetic xylose utilization pathway.

### Leakage in the modular design of the synthetic pathway

**Fig 5:**
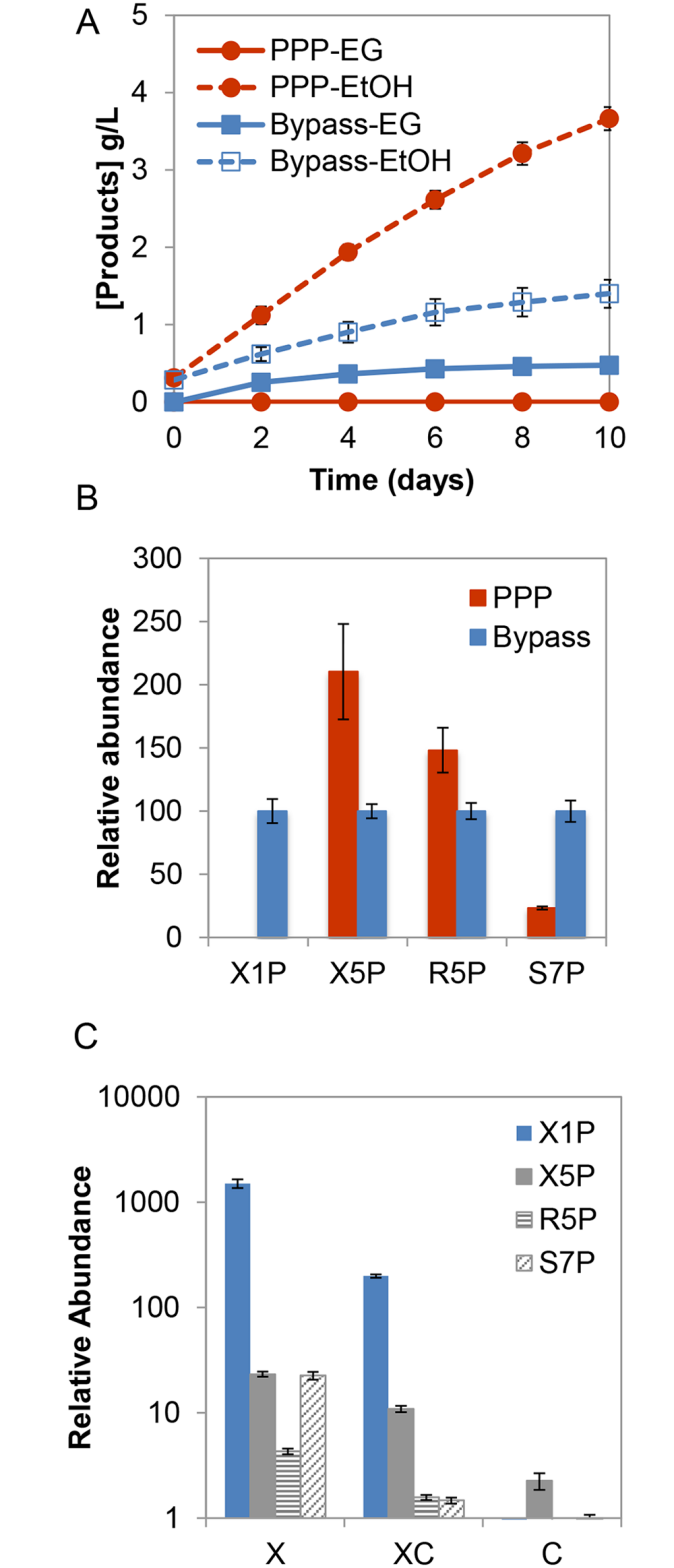
Product titers and relative abundance of metabolites in fermentations with the alternative pathway. The traditional xylose isomerase pathway (denoted as “PPP”) and the alternative xylose utilization pathway (*xks1Δ* XI-RnKHK-*FBA1*-CD denoted as “Bypass”) were compared. **(A)** Ethanol and ethylene glycol (EG) production. **(B)** Relative abundance of intracellular ᴅ-xylulose-1-phosphate (X1P), ᴅ-xylulose-5-phosphate (X5P), ᴅ-ribose-5-phosphate (R5P) and ᴅ-sedoheptulose-7-phosphate (S7P), after 4 days of fermentation. Values are normalized to 1 for levels of each compound observed in the Bypass strain. **(C)** Abundance of metabolites in the Bypass strain provided with xylose (X), a mixture of xylose and cellobiose (XC) and cellobiose only (C) are shown. Samples taken after 4 days of fermentation, and values are normalized to 1 for the levels of each compound observed in the cellobiose-only fermentation. In all three panels, error bars indicated standard errors, N = 5.

The three-step xylose utilization pathway for ethylene glycol biosynthesis is designed to bypass the central PPP (Fig 1). Product titers and the intracellular metabolites of the strain expressing the alternative synthetic pathway (*xks1Δ* XI-RnKHK-*FBA1-*CD strain) were compared to those of the traditional xylose utilization pathway (XI strain). For the synthetic pathway, the ethanol and EG titers were approximately 1.4 g/L and 0.48 g/L, respectively, resulting in a product molar ratio of 4:1 (ethanol:EG) (Fig 5A). As expected, metabolic profiling showed that X1P was only detected when the alternative pathway was expressed (Fig 5B). Notably, unique metabolites in the PPP—namely xylulose-5-phosphate (X5P), ribose-5-phosphate (R5P) and sedoheptulose-7-phosphate (S7P)—were observed at significant levels (Fig 5B), even though the new pathway was designed to bypass the PPP. While relative levels of X5P and R5P in the strain expressing the traditional pathway (XI strain) were 110% and 48% higher, S7P was 77% higher in the strain expressing the alternative pathway (Fig 5B).

In the *xks1Δ* background, there are three possible routes to X5P, R5P and S7P production in the PPP. First, reverse reactions involving fructose-6-phosphate (F6P), glyceraldehyde-3-phosphate (GADP), and erythrose-4-phosphate (E4P) (Fig 1) could result in X5P, R5P and S7P production. The steady-state flux distribution in the engineered strain could lead to buildups of X5P, R5P and S7P.

Second, it is possible that unannotated activities in *S*. *cerevisiae* are responsible for the conversion of X1P to X5P. To investigate the effect of a large buildup of X1P in the system, a strain expressing a 6xHis-tagged Fba1 was lysed and the Fba1 purified using a HisTrap column. The flowthrough of the lysate lacking Fba1 was incubated with X1P overnight. A decrease in X1P levels was observed in comparison to when no lysate was provided (S5A Fig). However, the expected product, X5P was not observed at the detection limit of the experiment (S6 Fig). Further mass spectrometry analysis was unable to identify the production of compounds with a molecular mass identical to that of X5P or xylulose. The resulting products could not be determined, potentially because of its rapid downstream reaction or the limitation of the mass spectrometry protocols. We also tested three endogenous proteins that might be responsible for X1P conversion to X5P—namely Pgm1, Pgm2 and Prm15. The purified enzymes were active in the conversion of glucose-1-phosphate to glucose-6-phosphate (S7 Fig) [24]. However, only purified Prm15 resulted in a decrease in X1P levels (S5B Fig). As with the crude lysates, mass spectrometry methods did not detect xylulose, X5P or compounds with similar masses to pentose monophosphates. In addition, tests of strains with individual deletions of *PGM1*, *PGM2*, or *PRM15* did not affect EG production titers, rates, or ethanol:ethylene glycol titer ratios appreciably (S5C and S5D Fig).

The third possible route for X5P, R5P and S7P production is from gluconeogenesis intermediates. Gluconeogenesis produces 6-carbon compounds from 3-carbon compounds under carbon starvation [25]. Beginning with 6-phosphogluconate, 6-carbon compounds can be converted to ribulose-5-phosphate and ribose 5-phosphate (R5P), X5P and S7P, respectively. To better understand the effect of upper glycolytic intermediates and the X1P synthetic pathway, cellobiose was supplied to the system in addition to xylose. The levels of X1P, X5P, R5P and S7P decreased in comparison to when xylose was provided as a sole carbon source (Fig 5C). This finding supports the hypothesis that PPP intermediates present in the X1P system may be a result of upper glycolytic intermediates produced by gluconeogenesis.

## Discussion

Although the PPP is the canonical route for pentose sugar utilization in microbes [26], the PPP in *S*. *cerevisiae* has not evolved to efficiently consume these sugars. We therefore devised an alternative route for xylose consumption (Fig 1). In our present design, three carbons in xylose can be directed to glycolysis in three linear steps, while the remaining two carbons can be converted to ethylene glycol, with a total loss of 1 carbon via CO_2_ for every molecule of xylose consumed (20% carbon loss) (Fig 1). In contrast, the traditional pathway requires 3 molecules of xylose to produce 5 molecules of ethanol accompanied by a loss of 5 CO_2_ (33.3% carbon loss) [27]. Thus when fully optimized, the alternative pathway in theory should be advantageous over the traditional pathway in terms of theoretical carbon loss. In this study, the theoretical carbon loss calculation was not present because the leakage problem detected in the synthetic pathway likely disrupted the stereochemistry of the reactions. Producing excess NAD^+^, this synthetic pathway could be coupled with NAD^+^ deficient metabolic pathways; for example, a cellobiose utilization pathway and 2,3-butandiol production pathways [28], to achieve an overall redox balance. Furthermore, the modular nature of this pathway may prove advantageous for understanding pentose utilization in *S*. *cerevisiae*. Here, we used ethylene glycol production from the alternative xylose utilization pathway as a phenotypic indicator of its metabolic efficiency, for two reasons. First, native *S*. *cerevisiae* strains do not synthesize EG. Thus, EG can be used as a readout for the efficiency of the alternative pentose utilization pathway. Second, EG is currently produced from ethylene derived from nonrenewable petrochemical resources [29]. The biosynthetic routes to EG from xylose were demonstrated in *Escherichia coli* via xylonate and 2-dehydro-3-deoxy-d-pentonate [30] and d-ribulose 1-phosphate [31,32]. With a rising global demand for EG, a biosynthetic pathway in *S*. *cerevisiae–*the preferred microbe for industrial fermentations–would prove beneficial for large-scale bioprocessing.

We found that *xks1Δ* was necessary for EG production (Fig 2). This is likely necessary at present, because the expression levels of enzymes within the synthetic pathway are suboptimal or unbalanced. By deleting the endogenous xylulokinase (*XKS1*), the major route for xylulose utilization via X5P and the PPP was removed, thereby forcing the intracellular xylulose to go through the alternative synthetic pathway. In comparison to the PPP, xylose consumption through the X1P pathway was slower (Fig 2A). This result implies that enzymes in the pathway downstream of xylose isomerase function suboptimally. Taking advantage of the 3-step linear fashion of the synthetic pathway, we found that overexpression of the downstream aldolase *FBA1* increased the ethylene glycol titer and accelerated xylose consumption, whereas overexpression of *GRE2* and/or *ADH1* resulted in limited improvements (Fig 3). This may be due to the fact that cleavage of X1P to DHAP and glycolaldehyde by Fba1 is thermodynamically unfavorable [33]. However, the negligible benefit of *GRE2* and/or *ADH1* overexpression was unexpected because glycolaldehyde is toxic to *S*. *cerevisiae* cells [16]. Therefore, in the present implementation, the enzymatic steps following xylulose formation but prior to glycolaldehyde production—namely ketohexokinase and aldolase—are likely to be the limiting steps in the new pathway (Fig 1).

Given that ketohexokinase requires ATP to phosphorylate xylulose early in the pathway, the system may face an ATP deficiency similar to the case of unbalanced glycolysis [34]. To test this model, a cellobiose utilization pathway was introduced to increase ATP equivalents available for xylose conversion without competing for xylose transport. Cellobiose fermentation is expected to provide the system with 3–4 ATPs for every molecule of cellobiose consumed [35], while only one net ATP was expected from the synthetic pathway per one molecule of xylose. When cellobiose was provided, the intracellular ATP and NADH increased by 42% and 104%, respectively, and the EG titer nearly tripled (Fig 4). Its synergistic effect with *FBA1* overexpression (Fig 4) also suggested that both ketohexokinase and aldolase are independent limiting steps of the pathway. In addition to providing excess ATP, cellobiose fermentation may also help with co-factor recycling. Its fermentation usually generates glycerol, potentially due to an excess of NADH that overflows from the acetaldehyde oxidation branch [22]. Since the synthetic xylose consumption pathway is NADH deficient, excess NADH from cellobiose fermentation may help keep the system balanced. This hypothesis is supported by a 15% decrease in glycerol titer (S3 Fig) observed in the cellobiose-xylose mixture in comparison to when cellobiose was provided as a sole carbon source. However, when formate was provided to the synthetic xylose utilization system, generating NADH by endogenous formate dehydrogenase (Fdh1) [23], xylose consumption improved while EG production worsened (S4 Fig). The small decrease in formate concentration implied limited activity of Fdh1. Increasing Fdh1 expression may provide more cellular NADH and may change the observed phenotypes. Taken together with the results when cellobiose was co-consumed, the NADH deficiency inherent in the pathway likely became more relevant when the system was not ATP limited.

The synthetic pathway is predicted to be fully modular and separate from the PPP. However, the high ratio of ethanol to EG (4:1 as opposed to the expected 1:1, Fig 1) suggested a leakage of the synthetic system into the PPP. Steady-state distributions suggested that unknown *S*. *cerevisiae* enzymes might be responsible for the leakage. However, our inability to find such an enzyme to date suggests that the leakage occurred through gluconeogenesis followed by production of PPP intermediates from 6-carbon compounds. Further biochemical studies of crude lysate fractions coupled with protein mass spectrometry should help identify the cause of X1P leakage.

The present results provide evidence that an alternative pentose utilization pathway in *S*. *cerevisiae* can partially bypass the X5P entry point and the PPP. This study showed the first evidence of X1P *in vivo* production, to the best of our knowledge. The synthetic pathway is simple and linear with three enzymatic steps to convert xylose to an intermediate for glycolysis. We envision that molecular and systems-level understanding of this new synthetic pathway could spur its broader application in alternative sugar utilization strategies.

## Materials and Methods

### Strains and Plasmids

*S*. *cerevisiae* D452-2 (*MATα*, *leu2*, *his3*, *ura3*, and *can1*) was used for the synthetic pathway construction. Plasmids and strains used in this work are described in S1 Table. The codon-optimized sequence for RnKHK is given at the end of the SI Materials and Methods. The In-Fusion HD Cloning Kit (Clontech) was used for all plasmid construction. Primers used are listed in S2 Table. Plasmids were transformed into the yeast strains using a standard lithium acetate yeast transformation protocol [36]. Transformants were selected on synthetic defined (SD) medium plates, which contained DOBA (MP Biomedicals, Santa Ana, CA) mixed with 2-fold appropriate CSM dropout mixture.

### Fermentation experiments

Single colonies from SD plates were selected and re-streaked. Re-streaked colonies were inoculated in Optimal Minimal Medium (oMM) [37] supplemented with 20 g/L of glucose to prepare seed cultures. For strains transformed with the plasmid encoding the cellobiose utilizing pathway (pCD), 20 g/L of cellobiose was provided in place of glucose. Seed cultures were harvested at mid- to late-exponential phase and washed twice with sterile water. Washed seed cultures were inoculate at an initial OD600 of 10–20 in 150 mL serum flasks containing 40 mL of media. The flasks were closed with butyl rubber stoppers, sealed with aluminum crimps, and purged with nitrogen gas to obtain strict anaerobic fermentations. The fermentation media contained oMM and 0.1 M 2-(*N*-morpholino)ethanesulfonic acid (MES), pH 6.0 supplemented with 40 g/L xylose, and/or 80 g/L cellobiose. The flasks were incubated at 30°C, 220 rpm. All fermentations were performed with two biological replicates.

### Analytical Methods

Extracellular concentrations of xylose, cellobiose, xylitol, glycerol, ethylene glycol and ethanol were determined by high performance liquid chromatography on a Prominence HPLC (Shimadzu) equipped with Aminex HPX-87H 300x7.8 mm column. The column was eluted with 0.01 N of H_2_SO_4_ at a flow rate of 0.5 mL/min, 30°C.

Intracellular metabolites at the exponential phase of ethylene glycol production (t = 4 days) were quenched in 180 μL of 40:40:20 acetonitrile:methanol:water. Following the addition of 10 nmols of d3 serine (as an internal standard), the mixtures were vortexed and centrifuged at 13,000 rpm for 10 minutes. The supernatants were injected onto an Agilent 6460 QQQ LC-MS/MS and the chromatography was achieved by normal phase separation with a Luna NH_2_ column (Phenomenex) starting with 100% acetonitrile with a gradient to 100% 95:5 water acetonitrile. 0.1% formic acid or 0.2% ammonium hydroxide with 50 mM ammonium acetate was added to assist with ionization in positive and negative ionization mode, respectively. Data were quantified by integrating the area under the curve of each metabolite multiple reaction monitoring transition, compared to internal standard. Five biological replicates were used for each sample analyzed.

X1P, X5P and S7P were separated using a 1200 Series liquid chromatography instrument (Agilent Technologies, Santa Clara, CA). A 1 μL aliquot of the sample was injected onto an Agilent Eclipse XDB-C18 (2.1 mm i.d., 150 mm length, 3.5 μm particle size) column with a Zorbax SB-C8 (2.1 mm i.d., 12.5 mm length, 5 μm particle size) guard column and eluted at 25°C and a flow rate of 0.2 mL/min with the following gradient (modification from [38]): 15 min isocratic 100% buffer A (10 mM tributylamine/15 mM acetic acid), then in 15 min with a linear gradient to 60% buffer B (methanol), 2 min isocratic 60% B, then 10 min equilibration with 100% buffer A. The eluent from the column was introduced into a mass spectrometer for 25 minutes after the first 10 minutes. Mass spectrometry (MS) was performed on an LTQ XL ion trap instrument (Thermo Fisher Scientific, San Jose, CA) with an ESI source operated in negative ion mode. The MS settings were capillary temperature 350°C, ion spray voltage 4.5 kV, sheath gas flow: 60 (arbitrary units), auxiliary gas flow 10 (arbitrary units), sweep gas flow 5 (arbitrary units). For MS/MS product ion scan, the scan range was m/z 80 to m/z 300. The compounds X1P, X5P and R5P at m/z 229.1 and S7P at m/z 289.1 were isolated with an m/z 2 isolation width and fragmented with a normalized collision-induced dissociation energy setting of 35% and with an activation time of 30 ms and an activation Q of 0.250. The detection limits for X1P and X5P based on this method were approximately 1–5 μM, depending on the experiment (S6 Fig).

### Xylulose phosphate synthesis

d-xylulose-1-phosphate (X1P) and d-xylulose-5-phosphate (X5P) were synthesized using purified rat liver ketohexokinase (RnKHK) and *E*.*coli* xylulokinase (xylB), respectively. The N-terminally His_6_-tagged RnKHK (pNT/His-RnKHK, S1 Table) was expressed in D452-2 *xks1Δ* and purified using HisTrap^™^ HP (GE Healthcare Life Sciences) according to the provided protocol. *E*.*coli* xylulokinase was cloned into pET302 N-terminal his-tag construct (pET-xylB, S1 Table) expressed in BL21 Star (DE3), induced with 0.2 mM IPTG and purified using HisTrap^™^ HP column. The reactions comprised of 1 mM d-xylulose and 1 mM ATP, catalyzed by 1 μM purified enzyme in 1X PBS buffer, were incubated at 30°C and 37°C overnight for the purified RnKHK and xylB, respectively. Formation of the products was verified by mass spectrometry according to the protocol previously described.

### Mutase purifications and activity assays

*PGM1*, *PGM2* and *PRM15* were cloned from *S*. *cerevisiae* D452-2 genomic DNA into plasmid pRS423 to create N-terminal His_6_-tag fusion constructs. The constructs were transformed and expressed in strain D452-2 in oMM media supplied with 20 g/L glucose. The cells were lysed and purified using HisTrap^™^ HP (GE Healthcare Life Sciences) according to standard protocols. Purified enzymes or crude cell lysates were incubated with 1 mM X1P synthesized as previously described in 1X phosphate buffered saline (PBS), pH 7.4 at 30°C. The reactions were stopped by addition of 0.1 M NaOH solution.

## Supporting Information

S1 Fig *In vivo* comparison of xylose isomerase (XI) and xylose reductase(XR)/xylitol dehydrogenase (XDH) conversions of xylose to xylulose in wild-type *XKS1* and *xks1*Δ strains expressing RnKHK. Strains were provided 40 g/L xylose as a sole carbon source under anaerobic conditions. (A) OD600 values, concentrations of (B) xylose (C) xylitol, (D) glycerol (E) ethylene glycol and (F) ethanol are shown. Error bars indicated standard errors, N = 2. NC and EG denote negative control and ethylene glycol, respectively.
(PDF)

S2 Fig Effect of overexpression of *FBA1*, *ADH1* and/or *GRE2*. *xks1Δ* XI-RnKHK was used as the background strain for the overexpression comparisons. (A) OD600 values, concentrations of (B) xylose (C) xylitol, (D) glycerol (E) ethylene glycol and (F) ethanol are shown. Error bars indicated standard errors, N = 2. NC and EG denote negative control and ethylene glycol, respectively.
(PDF)

S3 Fig Effect of cellobiose and xylose co-utilization. Strain *xks1Δ* XI-RnKHK-*FBA1*-CD was supplied with 80 g/L cellobiose, 40 g/L xylose or the mixture of 80 g/L cellobiose and 40 g/L xylose, denoted as C, X and XC, respectively. (A) OD600 values, concentrations of (B) cellobiose, (C) xylose (D) xylitol, (E) glycerol and (F) ethanol are shown. Error bars indicated standard errors, N = 5.
(PDF)

S4 Fig Effect of formate addition to the fermentation broth. (A) Crude lysate activities of formate dehydrogenase (Fdh1p) and butanediol dehydrogenase (Bdh1p). 50 mM formate or 50 mM 2,3-BDO and 1 mM NAD+ were incubated at 30°C in 50 mM MES, pH 6.0 with or without cell lysates. NADH was measured spectrophotometrically using absorption at 340 nm and compared to the NADH calibration curve. (B) xylose (C) ethylene glycol (D) ethanol (E) xylitol (F) glycerol and (G) formate concentrations of anaerobic fermentation provided with xylose and varied concentrations of formate (denoted as F) are reported. Error bars indicated standard errors, N = 2.
(PDF)

S5 Fig Activities of crude lysates and purified enzymes on d-xylulose-1-phosphate. **(A)** Relative abundance of X1P and X5P of reactions catalyzed by crude lysates from the traditional xylose utilization pathway (denoted as PPP) and the alternative pathway (*xks1Δ* XI-RnKHK-*FBA1*-CD denoted as Bypass), providing X1P as a substrate incubated overnight. **(B)** Relative abundance of X1P of reactions catalyzed by purified Pgm1p, Pgm2p, Prm15p providing X1P as a substrate and incubated for 1 hour. **(C)** ethylene glycol (EG) and **(D)** ethanol concentrations of fermentation systems with *pgm1*, *pgm2* and *prm15* deletion backgrounds in addition to *xks1* deletion expressing XI-RnKHK-*FBA1*-CD. Error bars indicated standard errors, N = 2.
(PDF)

S6 Fig Signal sensitivities of xylulose 5-phosphate (X5P) and xylulose 1-phosphate (X1P) detected by mass spectrometry. Experiments were carried out in triplicate. Error bars indicated standard errors, N = 3.
(PDF)

S7 Fig Purification of the three mutases—Pgm1p, Pgm2p and Prm15p—and verification of their activities. Purified mutases **(A)** Pgm1p, **(B)** Pgm2p and **(C)** Prm15p were shown to have expected molecular weights of 64.1, 64.1 and 72.1 kDa, respectively (arrows). **(D)** Verification of activities of the purified mutases. Activities of purified Pgm1p, Pgm2p and Prm15p were tested by incubation with 1 mM glucose 1-phosphate (G1P) in 1X PBS, pH 7.4 buffer at 30°C. The reactions were stopped with 0.1 M NaOH after 1 hour. Glucose 6-phosphate (G6P) was detected when the purified mutases were included in the reactions. G6P was not detected in the control reaction with no enzyme present. Pgm1p, Pgm2p and Prm15p are known to catalyze G1P and G6P conversion. The chromatograms are shown with 2 minute offsets.
(PDF)

S1 Table Plasmids and strains used in this study. (DOCX)

S2 Table Primers used in this study. (DOCX)
