# Supplementary figures and images for: Bypassing the Pentose Phosphate Pathway: Towards Modular Utilization of Xylose
Source: PLoS One. 2016 Jun 23;11(6):e0158111. doi: 10.1371/journal.pone.0158111 (PMC4918971; doi:10.1371/journal.pone.0158111)

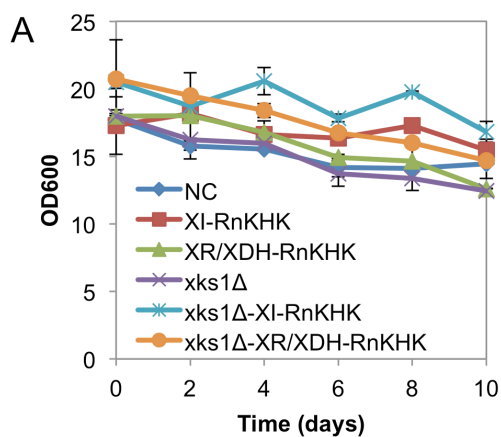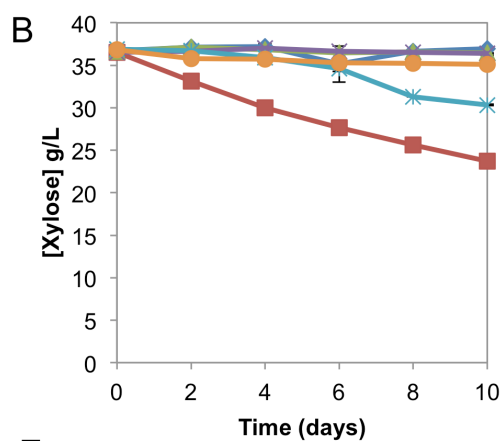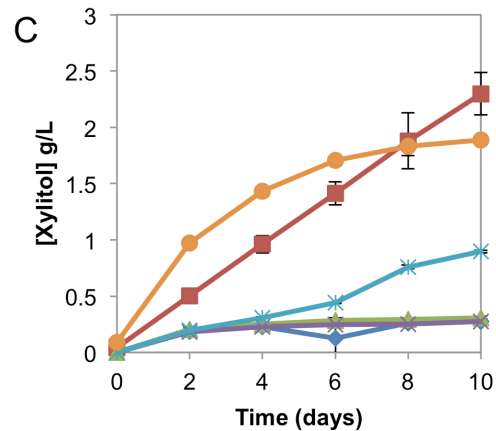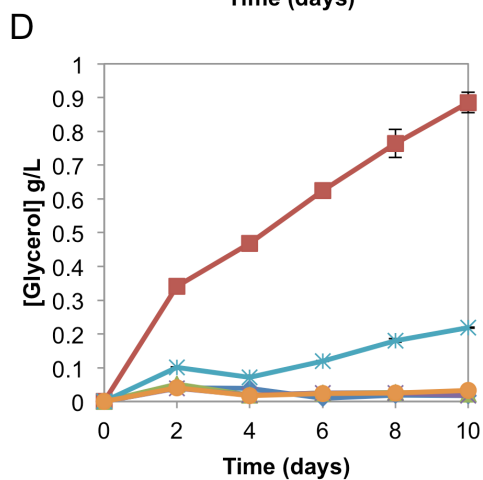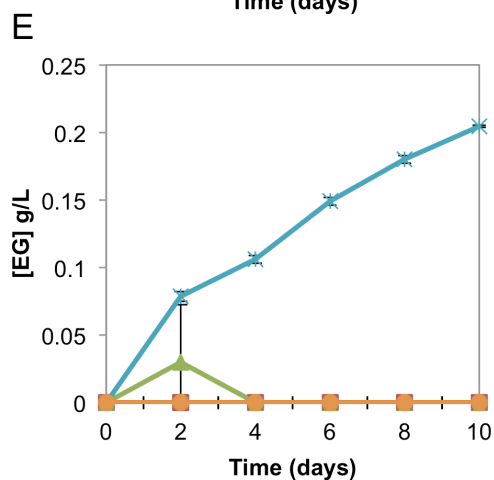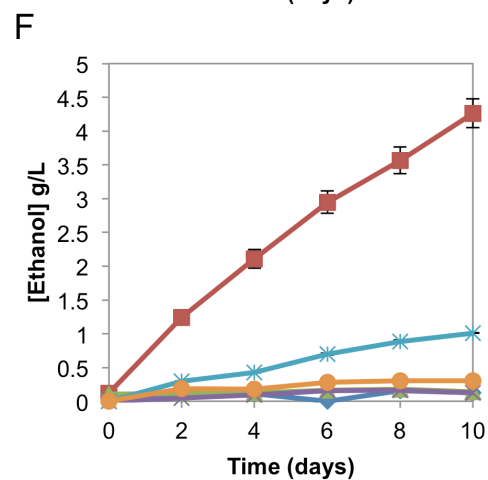

Supplement: S1 Fig — Strains were provided 40 g/L xylose as a sole carbon source under anaerobic conditions. (A) OD600 values, concentrations of (B) xylose (C) xylitol, (D) glycerol (E) ethylene glycol and (F) ethanol are shown. Error bars indicated standard errors, N = 2. NC and EG denote negative control and ethylene glycol, respectively. (PDF) [file pone.0158111.s001.pdf]

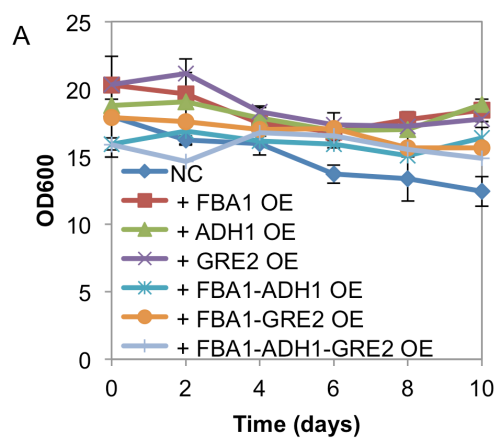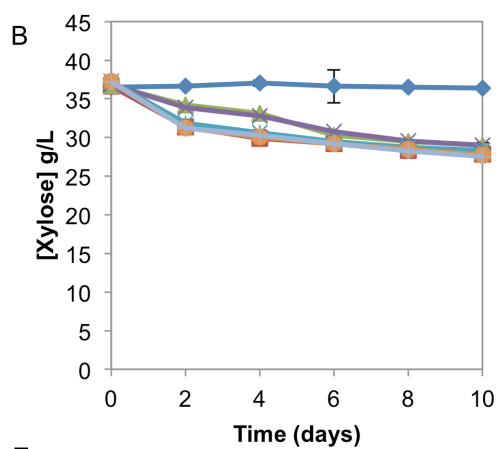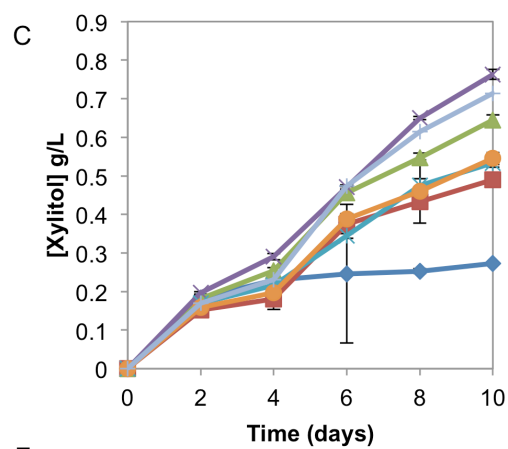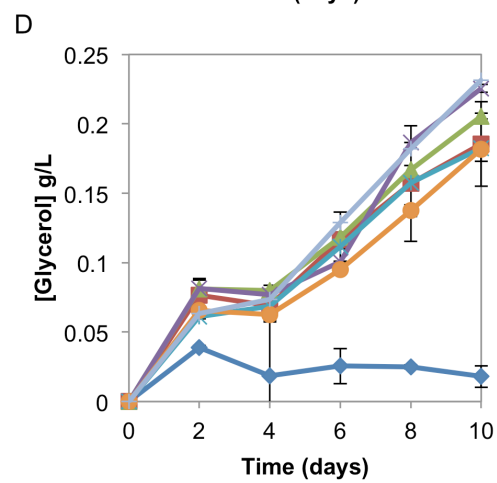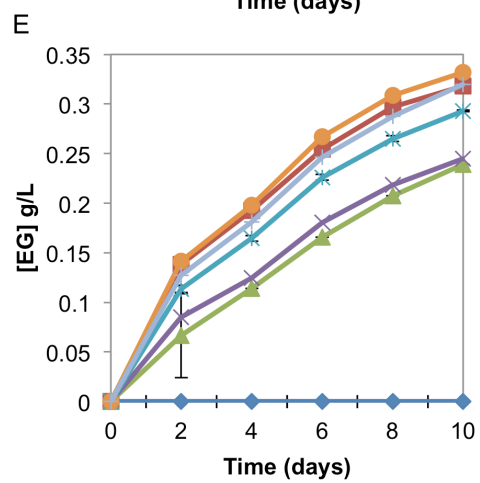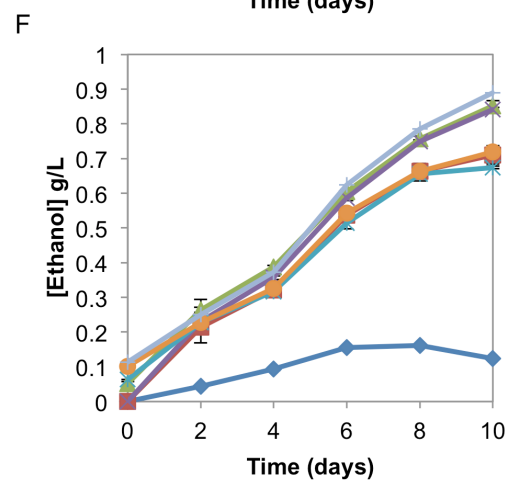

Supplement: S2 Fig — xks1Δ XI-RnKHK was used as the background strain for the overexpression comparisons. (A) OD600 values, concentrations of (B) xylose (C) xylitol, (D) glycerol (E) ethylene glycol and (F) ethanol are shown. Error bars indicated standard errors, N = 2. NC and EG denote negative control and ethylene glycol, respectively. (PDF) [file pone.0158111.s002.pdf]

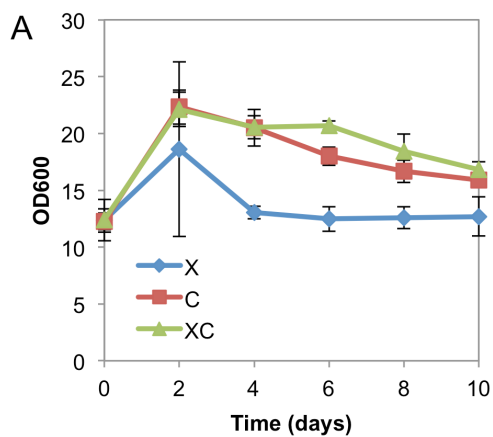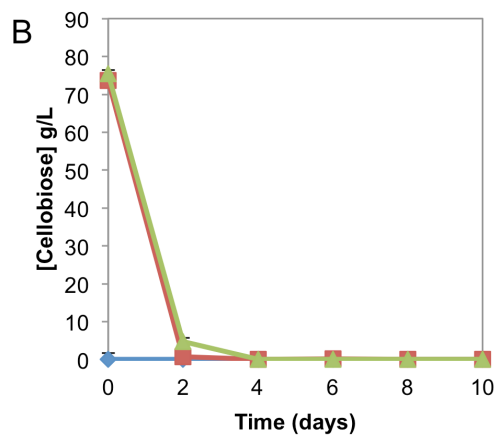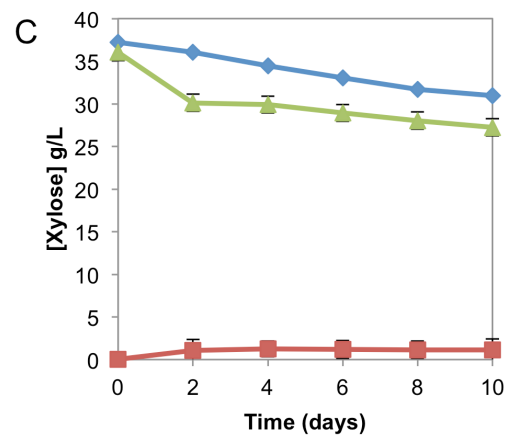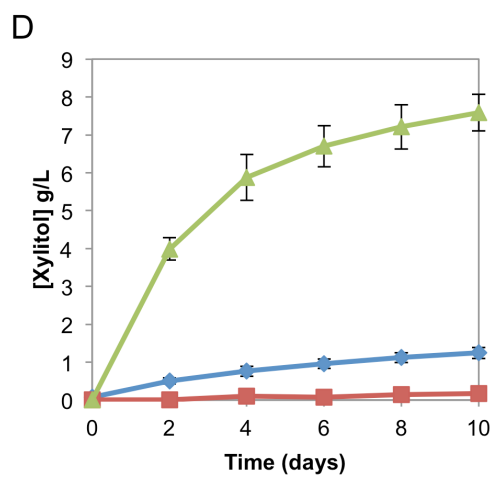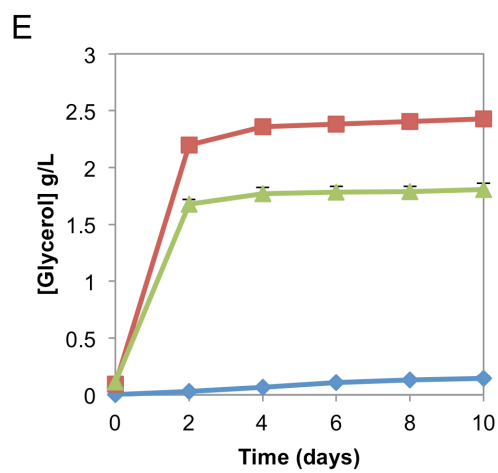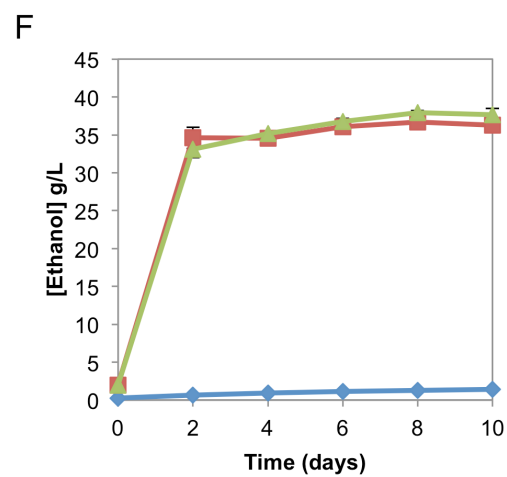

Supplement: S3 Fig — Strain xks1Δ XI-RnKHK-FBA1-CD was supplied with 80 g/L cellobiose, 40 g/L xylose or the mixture of 80 g/L cellobiose and 40 g/L xylose, denoted as C, X and XC, respectively. (A) OD600 values, concentrations of (B) cellobiose, (C) xylose (D) xylitol, (E) glycerol and (F) ethanol are shown. Error bars indicated standard errors, N = 5. (PDF) [file pone.0158111.s003.pdf]

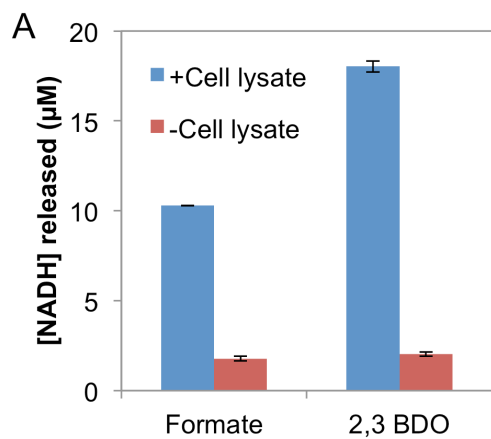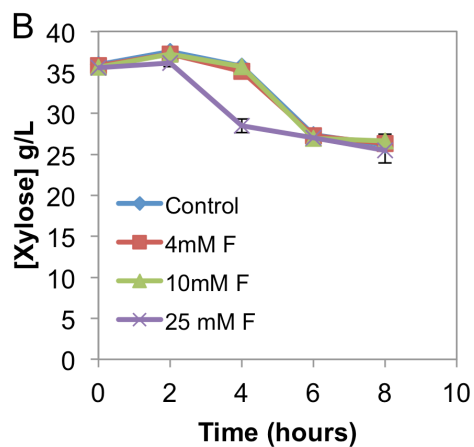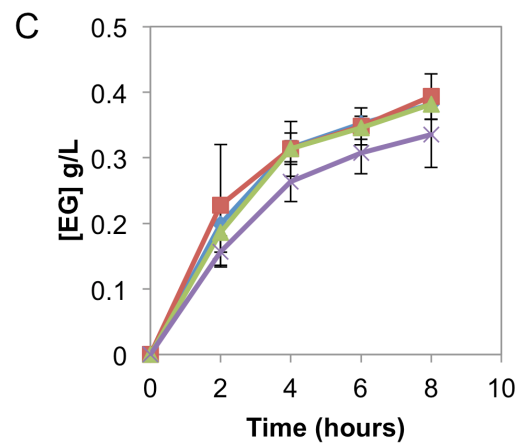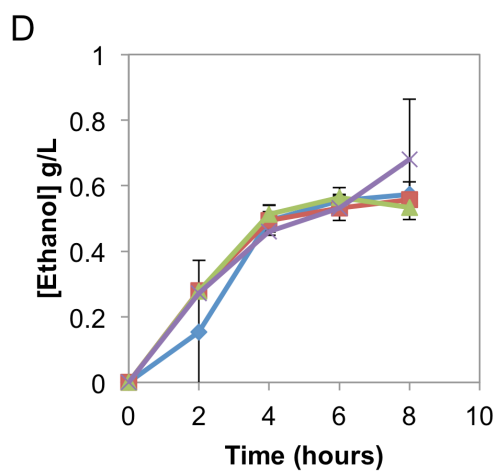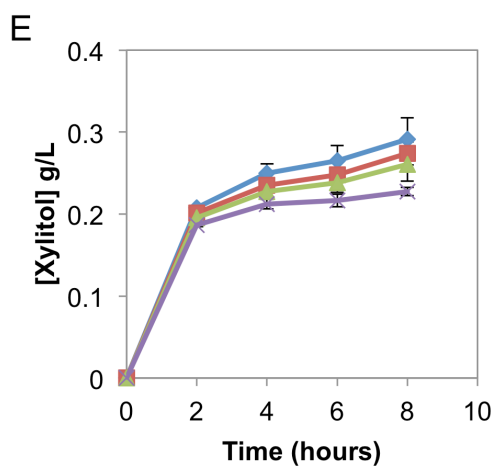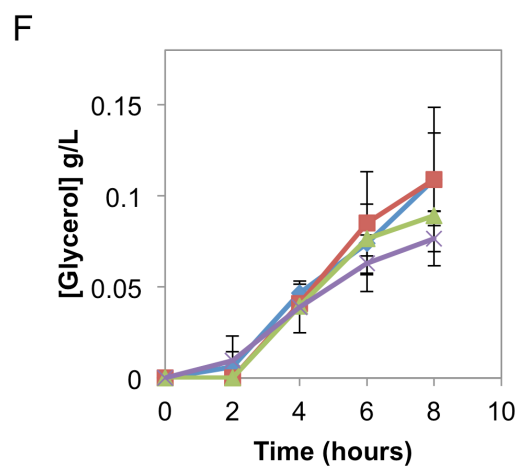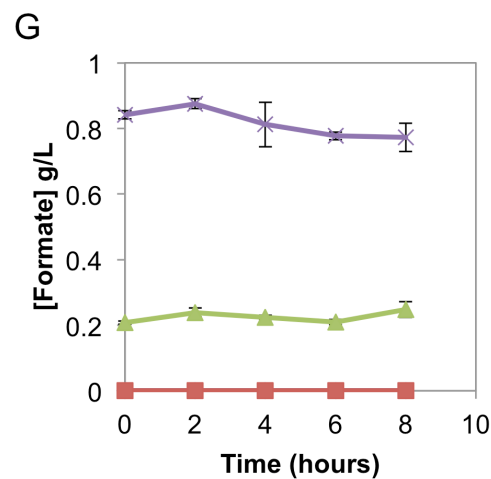

Supplement: S4 Fig — (A) Crude lysate activities of formate dehydrogenase (Fdh1p) and butanediol dehydrogenase (Bdh1p). 50 mM formate or 50 mM 2,3-BDO and 1 mM NAD+ were incubated at 30°C in 50 mM MES, pH 6.0 with or without cell lysates. NADH was measured spectrophotometrically using absorption at 340 nm and compared to the NADH calibration curve. (B) xylose (C) ethylene glycol (D) ethanol (E) xylitol (F) glycerol and (G) formate concentrations of anaerobic fermentation provided with xylose and varied concentrations of formate (denoted as F) are reported. Error bars indicated standard errors, N = 2. (PDF) [file pone.0158111.s004.pdf]

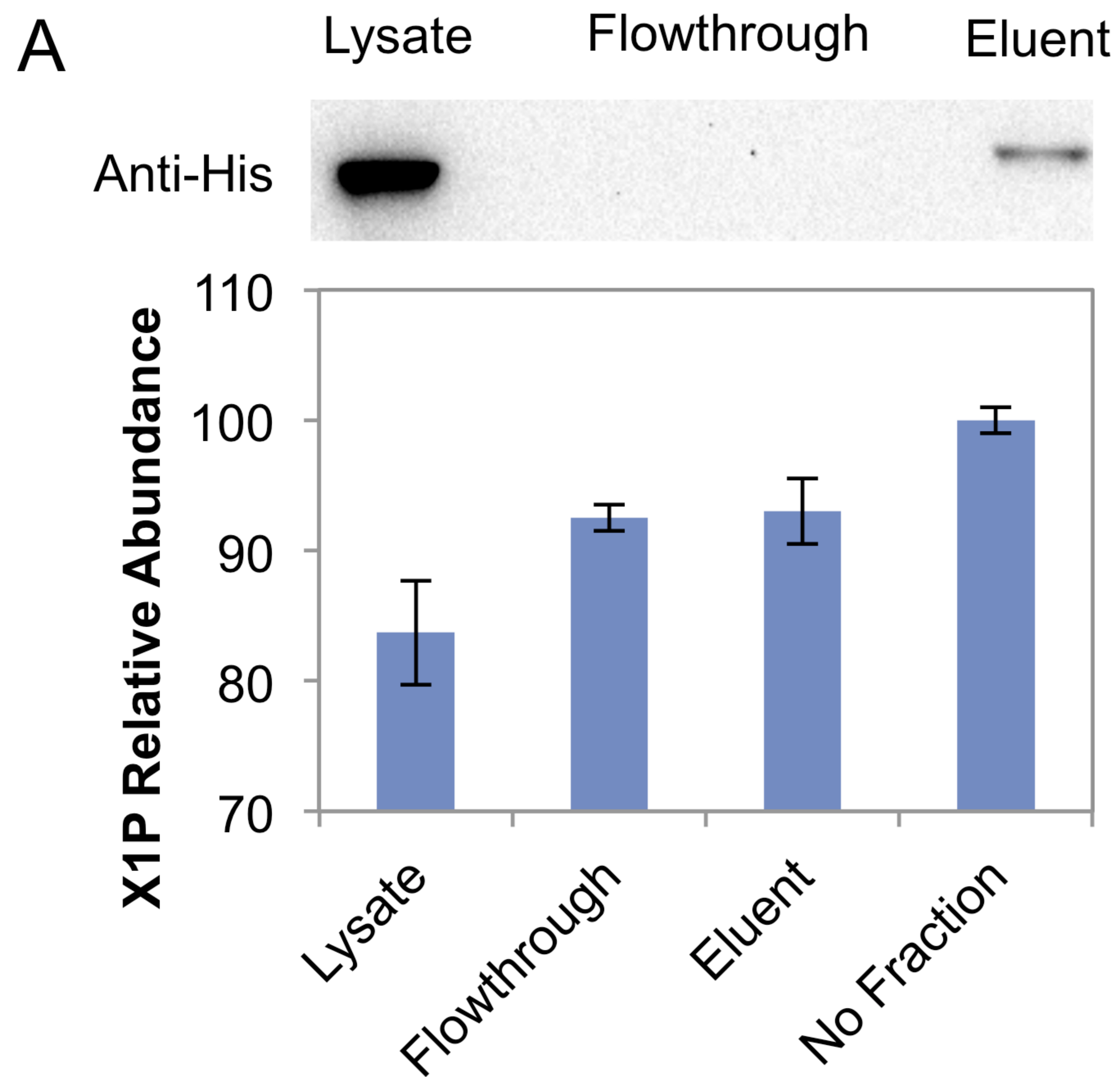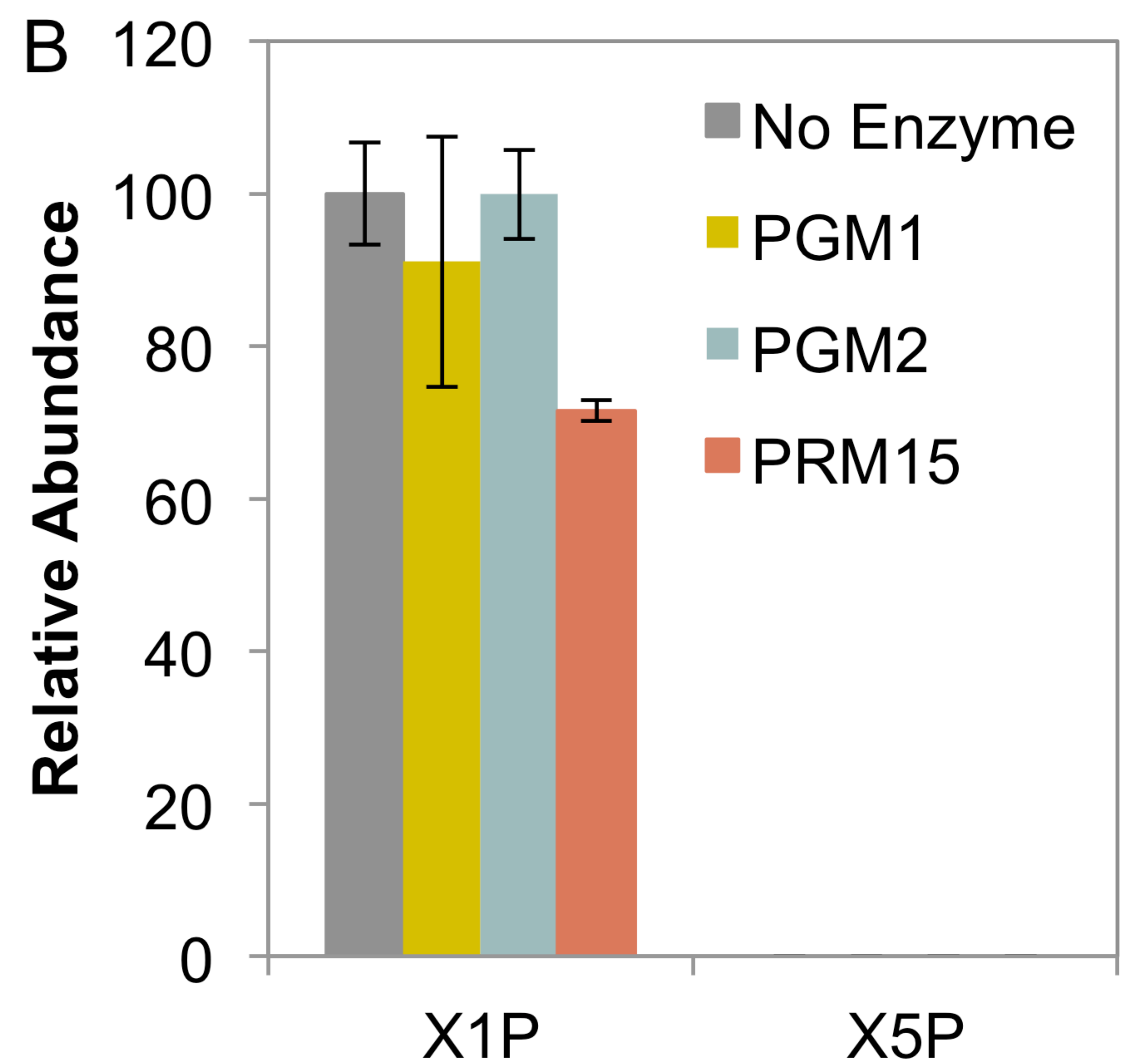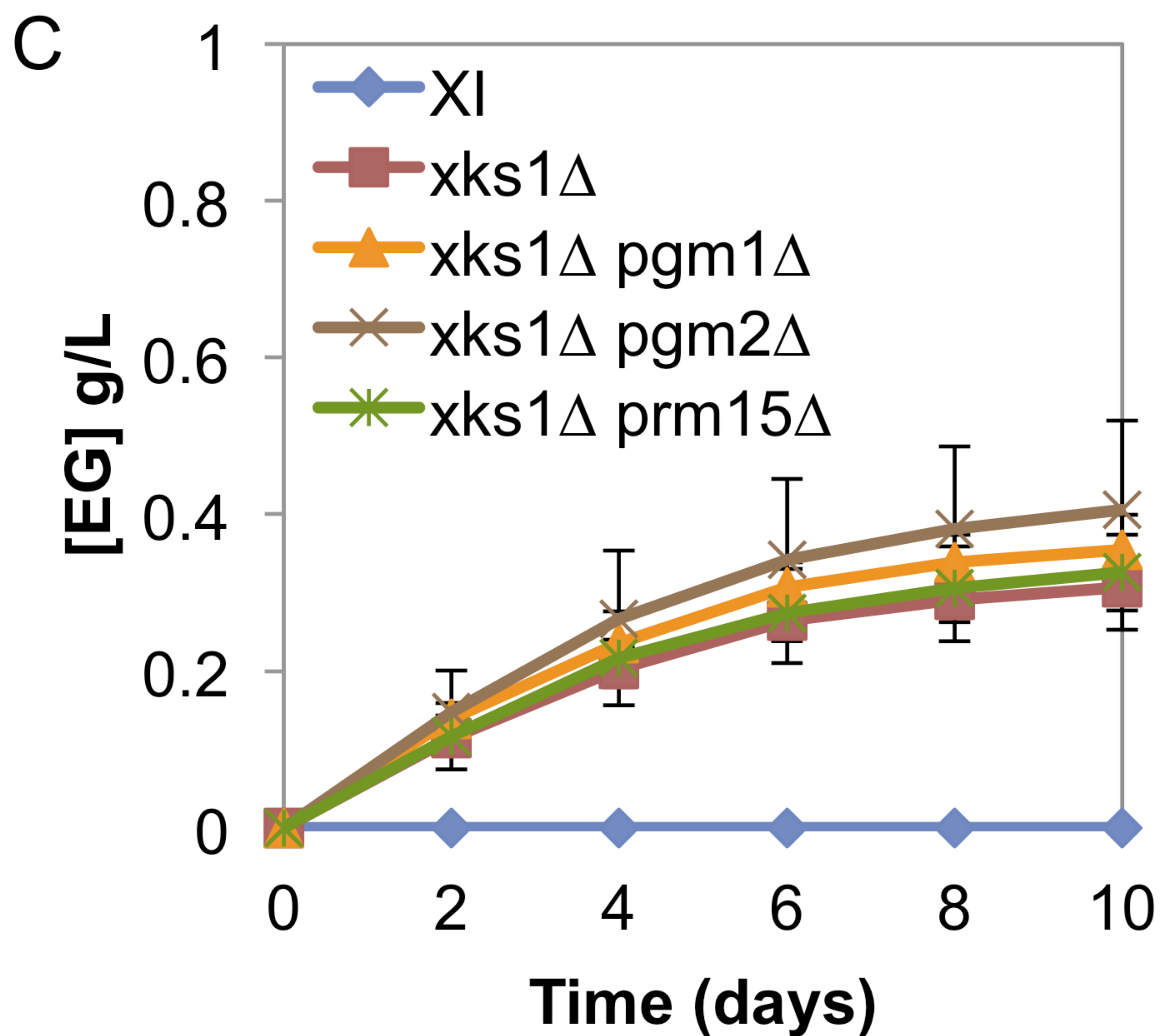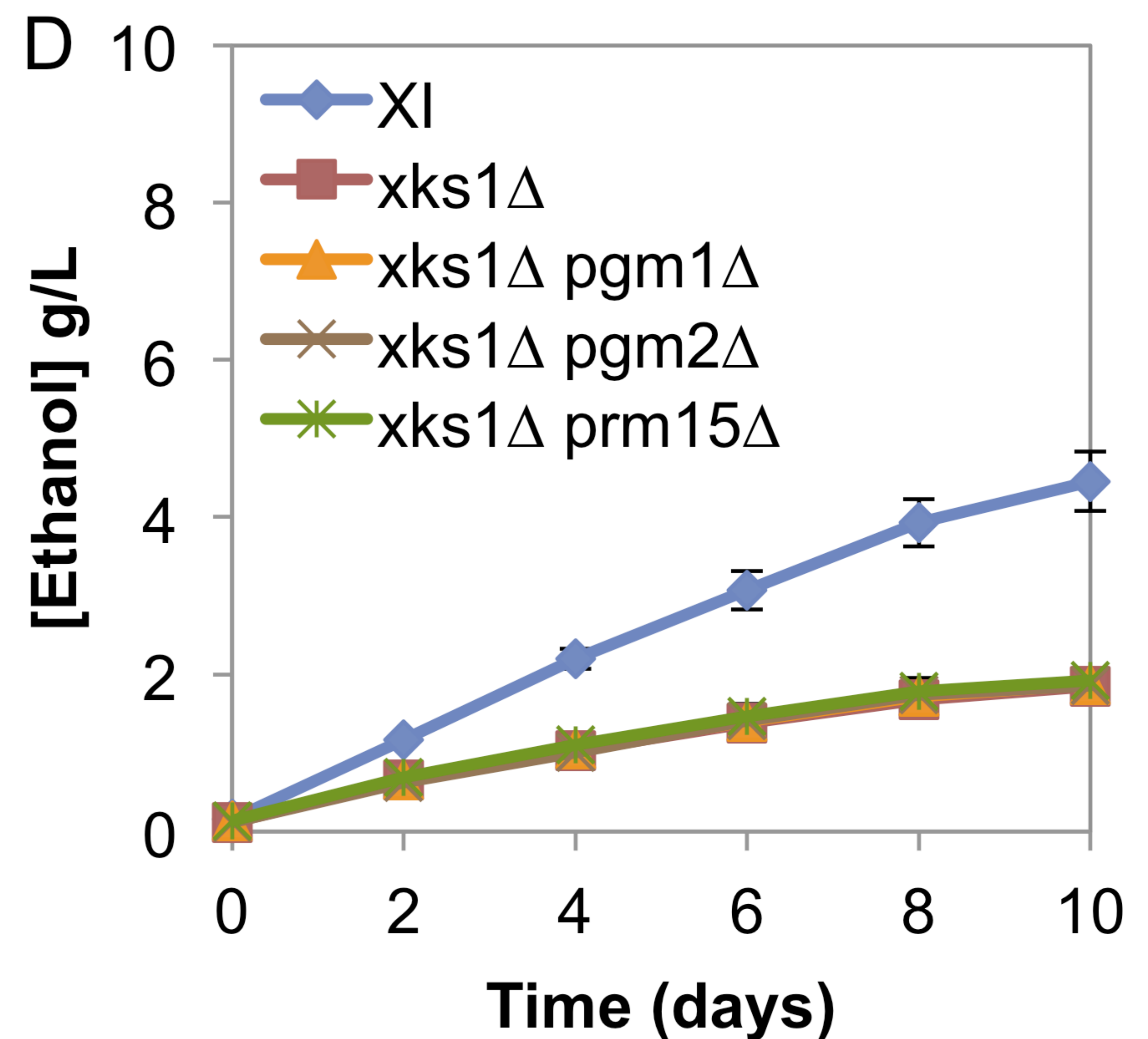

Supplement: S5 Fig — (A) Relative abundance of X1P and X5P of reactions catalyzed by crude lysates from the traditional xylose utilization pathway (denoted as PPP) and the alternative pathway (xks1Δ XI-RnKHK-FBA1-CD denoted as Bypass), providing X1P as a substrate incubated overnight. (B) Relative abundance of X1P of reactions catalyzed by purified Pgm1p, Pgm2p, Prm15p providing X1P as a substrate and incubated for 1 hour. (C) ethylene glycol (EG) and (D) ethanol concentrations of fermentation systems with pgm1, pgm2 and prm15 deletion backgrounds in addition to xks1 deletion expressing XI-RnKHK-FBA1-CD. Error bars indicated standard errors, N = 2. (PDF) [file pone.0158111.s005.pdf]

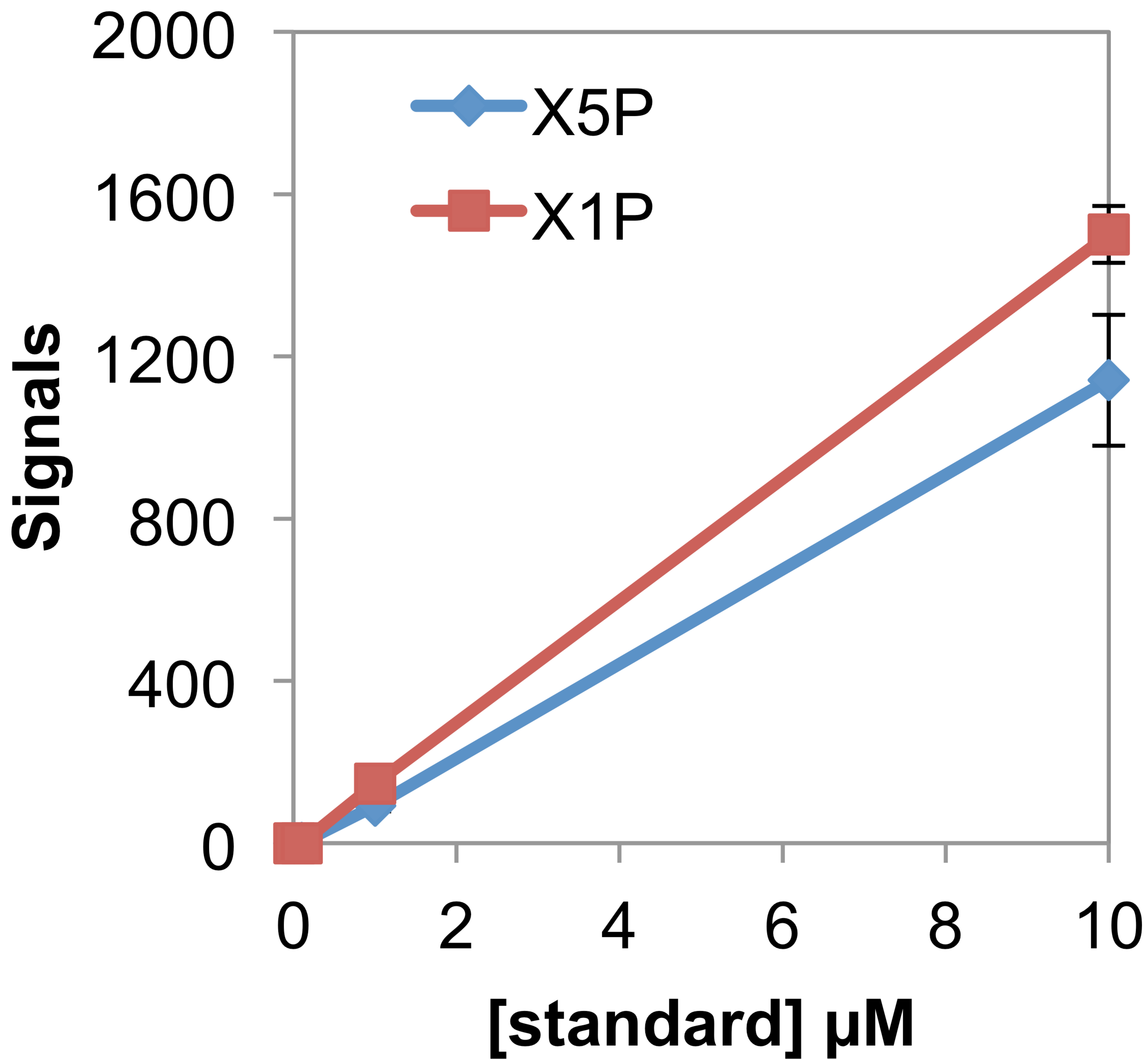

Supplement: S6 Fig — Experiments were carried out in triplicate. Error bars indicated standard errors, N = 3. (PDF) [file pone.0158111.s006.pdf]

A

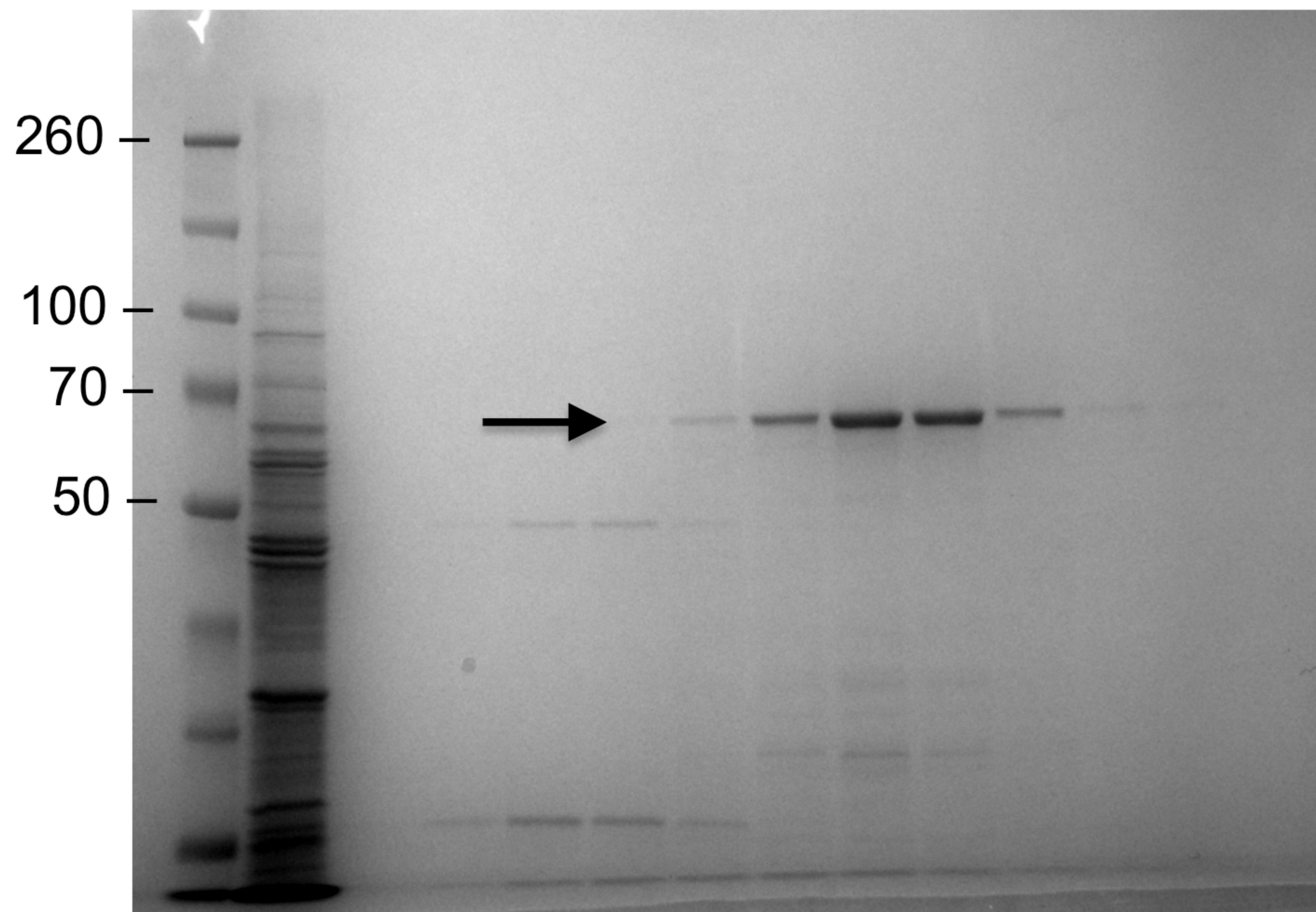

B

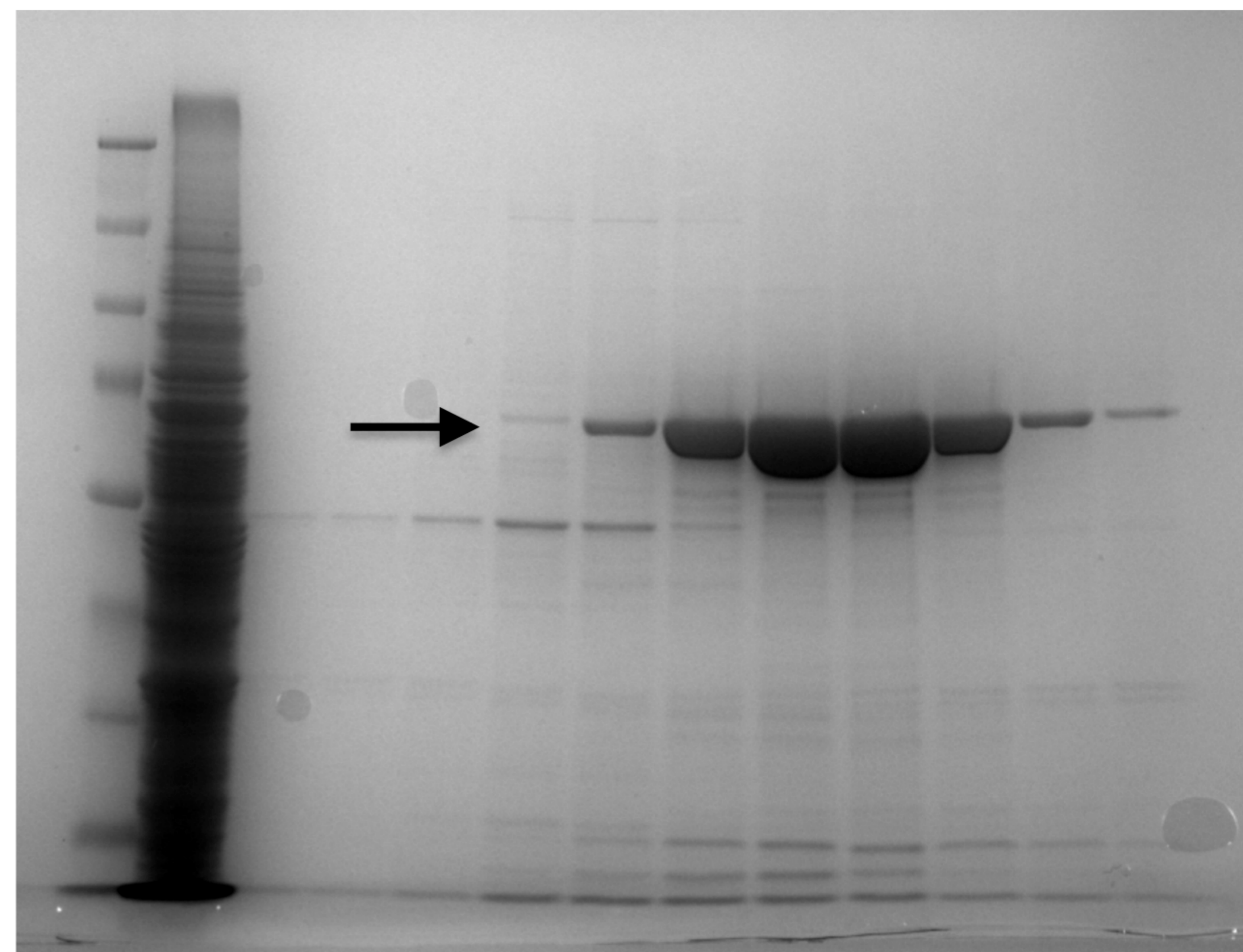

C

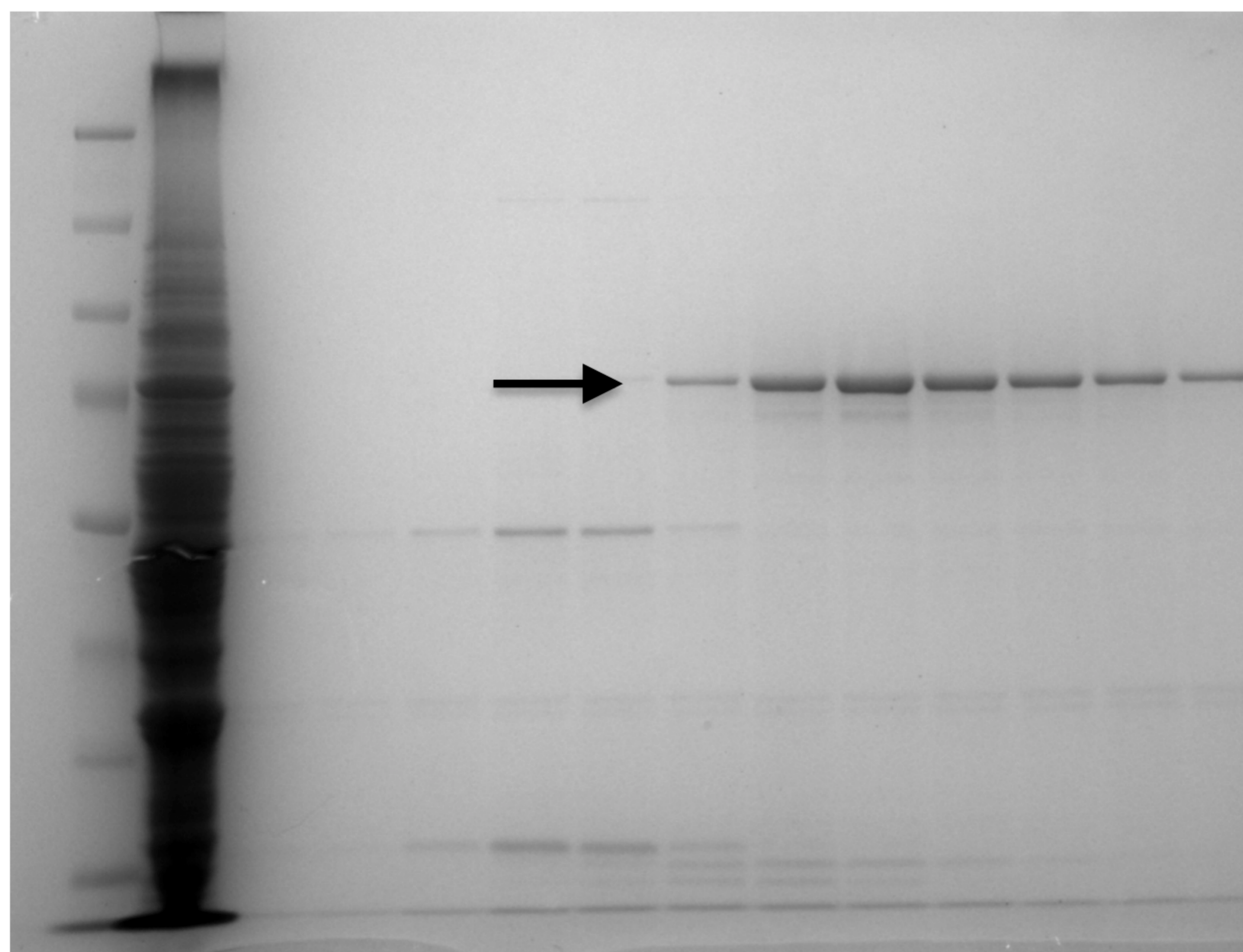

D

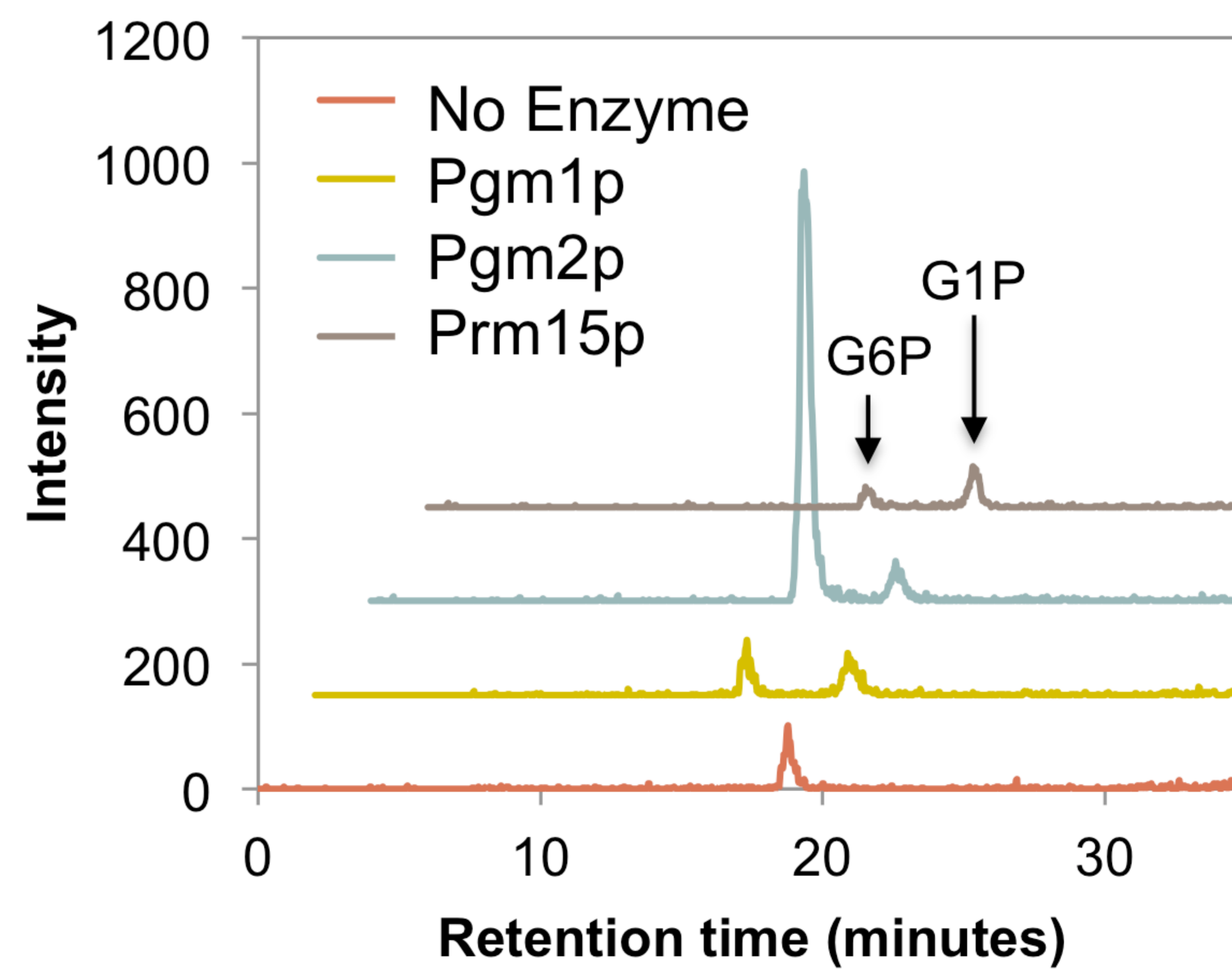

Supplement: S7 Fig — Purified mutases (A) Pgm1p, (B) Pgm2p and (C) Prm15p were shown to have expected molecular weights of 64.1, 64.1 and 72.1 kDa, respectively (arrows). (D) Verification of activities of the purified mutases. Activities of purified Pgm1p, Pgm2p and Prm15p were tested by incubation with 1 mM glucose 1-phosphate (G1P) in 1X PBS, pH 7.4 buffer at 30°C. The reactions were stopped with 0.1 M NaOH after 1 hour. Glucose 6-phosphate (G6P) was detected when the purified mutases were included in the reactions. G6P was not detected in the control reaction with no enzyme present. Pgm1p, Pgm2p and Prm15p are known to catalyze G1P and G6P conversion. The chromatograms are shown with 2 minute offsets. (PDF) [file pone.0158111.s007.pdf]
