# Supplementary material for: Bypassing the Pentose Phosphate Pathway: Towards Modular Utilization of Xylose
Source: PLoS One. 2016 Jun 23;11(6):e0158111. doi: 10.1371/journal.pone.0158111 (PMC4918971; doi:10.1371/journal.pone.0158111)
Supplement: S1 Table — (DOCX) [file pone.0158111.s008.docx]

**S1 Table. Plasmids and strains used in this study**

| **Plasmids/** | **Characterization** | **Reference** |
| --- | --- | --- |
| **Strains** |  |  |
| pRS313 | pRS313 | ATCC® 77142 |
| pRnKHK | pRS313-P*_FBA1_*-RnKHKopt-T*_CYC1_* | This study |
| pNT/his-RnKHK | pRS313-P*_FBA1_*-NT/his-RnKHKopt-T*_CYC1_* | This study |
| p1 | pRS313-P*_TDH3_*-RnKHKopt-T*_CYC1_* | This study |
| pRnKHK-FBA1 | pRS313-P*_FBA1_*-RnKHKopt-T*_CYC1_-*P*_FBA1_*-*FBA1*-T*_FBA1_* | This study |
| pSpKHK-FBA1 | pRS313-P*_FBA1_*-SpKHK-T*_CYC1_-*P*_FBA1_*-*FBA1*-T*_FBA1_* | This study |
| pNcKHK-FBA1 | pRS313-P*_FBA1_*-NcKHK-T*_CYC1_-*P*_FBA1_*-*FBA1*-T*_FBA1_* | This study |
| pRS315 | pRS315 | ATCC® 77144  Courtesy of Matthew Shurtleff |
| p2 | pRS424-P*_TEF1_*-BsXI-T*_CYC1_* | (5) |
| pXI | pRS315-P*_TEF1_*-BsXI-T*_CYC1_* | This study |
| pRS316 | pRS316 | ATCC® 77145 |
| p3 | pRS316-P*_CCW12_*-T*_CYC1_* | This study |
| pADH1 | pRS316-P*_CCW12_*-*ADH1*-T*_CYC1_* | This study |
| pGRE2 | pRS316-P*_CCW12_*-*GRE2*-T*_CYC1_* | This study |
| pADH1-GRE2 | pRS316-P*_CCW12_*-*ADH1*-T*_CYC1_-*P*_CCW12_*-*GRE2*-T*_CYC1_* | This study |
| pET-xylB | pET302-NT/his-xylB | This study |
| pCD | pRS316-P*_TDH3_*-CDT1-eGFP-T*_CYC1_-*P*_CCW12_*-gh1-1opt-T*_CYC1_* | Unpublished data (Lin *et al.,* 2014) |
| pRS423-NT/his-PGM1 | pRS423-NT/6xhis- P*_TEF1_*-PGM1-T*_CYC1_* | This study |
| pRS423-NT/his-PGM2 | pRS423-NT/6xhis- P*_TEF1_*-PGM2-T*_CYC1_* | This study |
| pRS423-NT/his-PRM15 | pRS423-NT/6xhis- P*_TEF1_*-PRM15-T*_CYC1_* | This study |
| S1 | *S. cerevisiae* D452-2 *MATalpha, leu2, his3, ura3,* and *can1*) | (6) |
| S2 | S1 *xks1Δ*::KanMX | This study |
| NC | S1 pRS313, pRS315, pRS316 | This study |
| XI-RnKHK | S1 pRnKHK, pXI, pRS316 | This study |
| xks1Δ | S2 pRS313, pRS315, pRS316 | This study |
| xks1Δ-XI-RnKHK | S2 pRnKHK, pXI, pRS316 | This study |
| + FBA1 OE | S2 pRnKHK-FBA1, pXI, pRS316 | This study |
| + ADH1 OE | S2 pRnKHK, pXI, pADH1 | This study |
| + GRE2 OE | S2 pRnKHK, pXI, pGRE2 | This study |
| + FBA1-ADH1 OE | S2 pRnKHK-FBA1, pXI, pADH1 | This study |
| + FBA1-GRE2 OE | S2 pRnKHK-FBA1, pXI, pGRE2 | This study |
| + FBA1-ADH1-GRE2 OE | S2 pRnKHK-FBA1, pXI, pADH1-GRE2 | This study |
| S3 | S2 pRnKHK, pXI, pCD | This study |
| Bypass | S2 pRnKHK-FBA1, pXI, pCD | This study |
| PPP | S1 pXI, pRS313, pCD | This study |
| S6 | S2 pNT/his-RnKHK | This study |
| S7 | *E.coli* BL21 (DE3) pET-xylB | This study |
| pgm1Δ | S2 *pgm1Δ*::*nat*MX pRnKHK-FBA1, pXI, pCD | This study |
| pgm2Δ | S2 *pgm2Δ*::*nat*MX pRnKHK-FBA1, pXI, pCD | This study |
| prm15Δ | S2 *prm15Δ*::*nat*MX pRnKHK-FBA1, pXI, pCD | This study |
