# Supplementary material for: Bypassing the Pentose Phosphate Pathway: Towards Modular Utilization of Xylose
Source: PLoS One. 2016 Jun 23;11(6):e0158111. doi: 10.1371/journal.pone.0158111 (PMC4918971; doi:10.1371/journal.pone.0158111)
Supplement: S2 Table — (DOCX) [file pone.0158111.s009.docx]

**S2 Table. Primers used in this study**

| **Primers** | **Sequence (5'-3')** | **Application** |
| --- | --- | --- |
| 313_FBA1p_F_site1_F | cgcggtggcggccgcataacaatactgacagtactaaataattgc | pRnKHK construction |
| 313_FBA1p_R_site1 | TTCTTCCATTCTAGATTTGAATATGTATTACTTGGTTATGG |  |
| 313_RnKHK_frag_F | tctagaatggaagaaaagcaaattttg |  |
| 313_RnKHK_frag_R | GACATAACTACTAGTTTAGACGATACCATCGAAACC |  |
| 313_CYC1t_frag_F | actagtagttatgtcacgcttacattcac |  |
| 313_CYC1t_Frag_R | CAGCCCGGGGGATCCAGCTTGCAAATTAAAGCC |  |
| N-RnKHKtag_1 | agtaatacatattcaaatctagaatgcatcaccatcacc | pNT/his-RnKHK construction |
| N-RnKHKtag_2 | catcaccatcaccatcaccatcacgcgtctgcgtggtctcatccacaatttgaaaaagg |  |
| N-RnKHKtag_3 | CAAAATTTGCTTTTCTTCTGGTCCTTGGAACAGGACTTCGAGACCACCTTTTTCAAATTG |  |
| TDH3p_RnKHK_F_Frag | cagttcgagtttatcattatcaatactgc | p1  construction |
| TDH3p_RnKHK_R_Frag | TTTGTTTGTTTATGTGTGTTTATTCGAAAC |  |
| TDH3p_RnKHK_F_LV | acataaacaaacaaatctagaatggaagaaaagcaaattttgtg |  |
| TDH3p_RnKHK_R_LV | GATAAACTCGAACTGCCGCGGTGGAGCTCC |  |
| 313_site2_FBA_F | atcgataccgtcgacataacaatactgacagtactaaataattgc | pRnKHK-FBA1 construction |
| 313_site2_FBA_R | CAAAAGCTGGGTACCGCTATCAAAAACGATAGATCGATTAG |  |
| 313_site2_LV_F | ggtacccagcttttgttcc |  |
| 313_site2_LV_R | GTCGACGGTATCGATAAGC |  |
| SpKHK frag for pFBA1_F | taatacatattcaaaatgtcttcatacatcttgtttggtttgg | pSpKHK-FBA1 construction |
| SpKHK frag for pFBA1_R | GACATAACTACTAGTTTAAGGTAAAGGGAACTGCTTTTTG |  |
| 313_FBA1p_R_site1 | [same as above] |  |
| 313_CYC1t_frag_F | [same as above] |  |
| NCU03742 frag for pFBA1_F | taatacatattcaaaatggaaggaaggaaggaag | pNcKHK-FBA1 construction |
| NCU03742 frag for pFBA1_R | GACATAACTACTAGTTTACCATCCCTTAACAATCC |  |
| 313_FBA1p_R_site1 | [same as above] |  |
| 313_CYC1t_frag_F | [same as above] |  |
| 315_TEF1p_frag_F | agtggatcccccgggatagcttcaaaatgtttctactcc | pXI construction |
| 315_CYC1t_frag_R | GTCGACGGTATCGATGGCCGCAAATTAAAGC |  |
| pRS315-LV_F | atcgataccgtcgacctcg |  |
| pRS315-LV_R | CCCGGGGGATCCACTAG |  |
| pRS316-CCW12p-frag_F | agtggatcccccgggcaaagcaaaataaaagaaacttaatacg | p3 construction |
| pRS316-CCW12p-frag_R | ACTAAGCTTCTGCAGTATTGATATAGTGTTTAAGCGAATGACAG |  |
| pRS316-CYC1t-frag_F | ctgcagaagcttagttatgtcacgcttacattcac |  |
| pRS316-CYC1t-frag_R | GTCGACGGTATCGATAGCTTGCAAATTAAAGCCTTC |  |
| 316_CCW12p_ADH1_frag_F | aacactatatcaataatgtctatcccagaaactcaaaaag | pADH1 construction |
| 316_CYC1t_ADH1_frag_F | AAGCGTGACATAACTTTATTTAGAAGTGTCAACAACGTATCTACC |  |
| 316_LV_CYC1t_F | agttatgtcacgcttacattcacg |  |
| 316_LV_CCW12p_R | TATTGATATAGTGTTTAAGCGAATGACAG |  |
| 316_CCW12p_GRE2_frag_F | aacactatatcaataatgtcagttttcgtttcaggtgc | pGRE2 construction |
| 316_CCW12p_GRE2_frag_R | AAGCGTGACATAACTTTATATTCTGCCCTCAAATTTTAAAATTTG |  |
| 316_LV_CYC1t_F | [same as above] |  |
| 316_LV_CCW12p_R | [same as above] |  |
| 316_site2_CCW12p_frag_F | agtggatcccccgggcaaagcaaaataaaagaaacttaatacg | pADH1-GRE2 construction |
| 316_site2_CYC1t_frag_R | ATCGAATTCCTGCAGAGCTTGCAAATTAAAGCCTTC |  |
| 316_site2_LV_F | ctgcaggaattcgatatcaagc |  |
| 316_site2_LV_R | CCCGGGGGATCCACTAG |  |
| xyIB_F | gttctgttccagggtccgtatatcgggatagatcttgg | pET-xylB construction |
| xyIB_R | CGGATCCGATTATACCTACGCCATTAATGGCAG |  |
| pETm_F | taggtataatcggatccggctg |  |
| pETm_R | CGGACCCTGGAACAGAAC |  |
| 423-his-PGM1-F | atgcatcatcatcatcatcatggtggtggttcacttctaatagattctgtaccaac | pRS423-NT/his-PGM1  Construction |
| 423-his-PGM1-R | TAATTACATGACTCGAGTTACTATGTGCGGACTGTTGG |  |
| 423-his-LV-F | taactcgagtcatgtaattag |  |
| 423-his-LV-R | TGATGATGATGATGATGCATTGGATCCACTAGTTCTAG |  |
| 423-his-PGM2-F | atgcatcatcatcatcatcatggtggtggttcatttcaaattgaaacgg | pRS423-NT/his-PGM2  Construction |
| 423-his-PGM2-R | TAATTACATGACTCGAGTTATTAAGTACGAACCGTTG |  |
| 423-his-LV-F | [same as above] |  |
| 423-his-LV-R | [same as above] |  |
| 423-his-PRM15-F | atgcatcatcatcatcatcatggtggtggtttgcaaggaattttagaaacc | pRS423-NT/his-PRM15  Construction |
| 423-his-PRM15-R | TAATTACATGACTCGAGTTATCAAAATTTTGTAACTATATTCATTTCATC |  |
| 423-his-LV-F | [same as above] |  |
| 423-his-LV-R | [same as above] |  |
| xks1del_UP_F | aaatgcgtttatatatatatattccagtg | S2 construction |
| xks1del_UP_R | TAAAGTACTAATCTCATCCTCCTTTTG |  |
| xks1del_DOWN_F | aatatgtttgagataatttatcatgc |  |
| xks1del_DOWN_R | GCCTCTTGGGGTAACTTTATAG |  |
| KanMX_F | aaacaaaaggaggatgagattagtactttataggtctagagatctgtttagc |  |
| KanMX_R | CAGGGCATGATAAATTATCTCAAACATATTATTAAGGGTTCTCGAGAG |  |
| pgm1_up_F | caggcatttatactttgg | S2 *pgm1Δ*::*nat*MX |
| pgm1_up_R | TACTATTCAATTGAGTTGTTAG |  |
| natMX_F | tctctctctcatccactttcggttcaatagtgcaaaaataggtaactaacaactcaattgaatagtaaTAGGTCTAGAGATCTGTTTAGC |  |
| natMX_R | CTCTACATATAAATTAAGATCAGTTAAAACATCTTTGGAAAATTAGTGCTTGTTCAAGACCAATAATTATTAAGGGTTCTCGAGAGC |  |
| pgm1_down_F | tggtcttgaacaagc |  |
| pgm1_down_R | GTTTTCTGAATTACCACC |  |
| pgm2_up_F | attaaaaaaggtctaacatcc | S2 *pgm2Δ*::*nat*MX |
| pgm2_up_R | ACTGAGAAAGATTGGTTG |  |
| natMX_F | caataggataataagaagaagatcaaccaatctttctcagtaaaaaaagtaacaaaagttaacataacTAGGTCTAGAGATCTGTTTAGC |  |
| natMX_R | TTTCTTCTTTACCGTTAATATTCATTGAAAAAGGTGAAAATCATTAAGCCATTAGTAAATCATTCGTTATTAAGGGTTCTCGAGAGC |  |
| pgm2_down_F | cgaatgatttactaatggc |  |
| pgm2_down_R | GGATTCTTGAAGGATGC |  |
| prm15_up_F | gacaagaatatctggagg | S2 *prm15Δ*::  *nat*MX |
| prm15_up_R | TTTTTGAAATTGCAGCAG |  |
| natMX_F | attctttctcctttccccctaaatactcaacagtactctcgagccattagctgctgcaatttcaaaaaTAGGTCTAGAGATCTGTTTAGC |  |
| natMX_R | AGGTACAAACAACAAATATAATGGCATTCTTTAAGTCGCTTGGTCTTAATGTATAGGTTAAAATAGTAATTAAGGGTTCTCGAGAGC |  |
| prm15_down_F | tattttaacctatacattaagacc |  |
| prm15_down_R | CATTCTATTATGTTTCCTTCC |  |
